# Murine norovirus allosteric escape mutants mimic gut activation

**DOI:** 10.1128/jvi.00219-25

**Published:** 2025-05-12

**Authors:** Michael B. Sherman, Hong Q. Smith, Faith Cox, Christiane E. Wobus, Gillian C. Lynch, B. Montgomery Pettitt, Thomas J. Smith

**Affiliations:** 1 Department of Biochemistry and Molecular Biology, University of Texas Medical Branch at Galveston 198643, Galveston, Texas, USA; 2 Department of Microbiology and Immunology, University of Michigan Medical School 12266 , Ann Arbor, Michigan, USA; Cornell University Baker Institute for Animal Health, Ithaca, New York, USA

**Keywords:** norovirus, antibodies, escape, cryo-EM

## Abstract

Murine norovirus (MNV) undergoes large conformational changes in response to the environment. The T=3 icosahedral capsid is composed of 180 copies of ~58 kDa VP1 that has N-terminal (N), shell (S), and C-terminal protruding (P) domains. In phosphate-buffered saline, the P domains are loosely tethered to the shell and float ~15 Å above the surface. At conditions found in the gut (i.e., low pH with high metal ion and bile salt concentrations), the P domain rotates and drops onto the shell with intra P domain changes that enhance receptor interactions while blocking antibody binding. Two of our monoclonal antibodies (2D3 and 4F9) have broad strain recognition, and the only escape mutants, V339I and D348E, are located on the C’D’ loop and ~20 Å from the epitope. Here, we determined the cryo-EM structures of V339I and D348E at neutral pH +/–metal ions and bile salts. These allosteric escape mutants have the activated conformation in the absence of gut triggers. Since this conformation is not recognized by antibodies, it explains how these mutants evade antibody recognition. Dynamic simulations of the P domain further suggest that movement of the C’D’ loop may be the rate-limiting step in the conformational change and that V339I increases the motion of the A’B’/E’F’ loops compared to the wild-type (WT), facilitating the transition to the activated state. These findings have important implications for norovirus vaccine design since they uncover a form of the viral capsid that should lend superior immune protection against subsequent challenge by wild-type virus.

**IMPORTANCE:**

Immune protection from norovirus infection is notoriously transient in both humans and mice. Our results strongly suggest that this is likely because the “activated” form of the virus found in gut conditions is not recognized by antibodies created in the circulation. By reversibly presenting one structure in the gut and a completely different antigenic structure in circulation, the gut tissue can be infected in subsequent challenges, while extraintestinal organs are protected. We find here that allosteric escape mutants to the most broadly neutralizing antibodies thwart recognition by transitioning to the activated state without the need for gut triggers (i.e., bile, low pH, or metal ions). These findings are significant because it is now feasible to present the activated form of the virus to the immune system (for example, as a vaccine) to better protect the gut tissue for longer periods of time.

## INTRODUCTION

**Fig 1:**
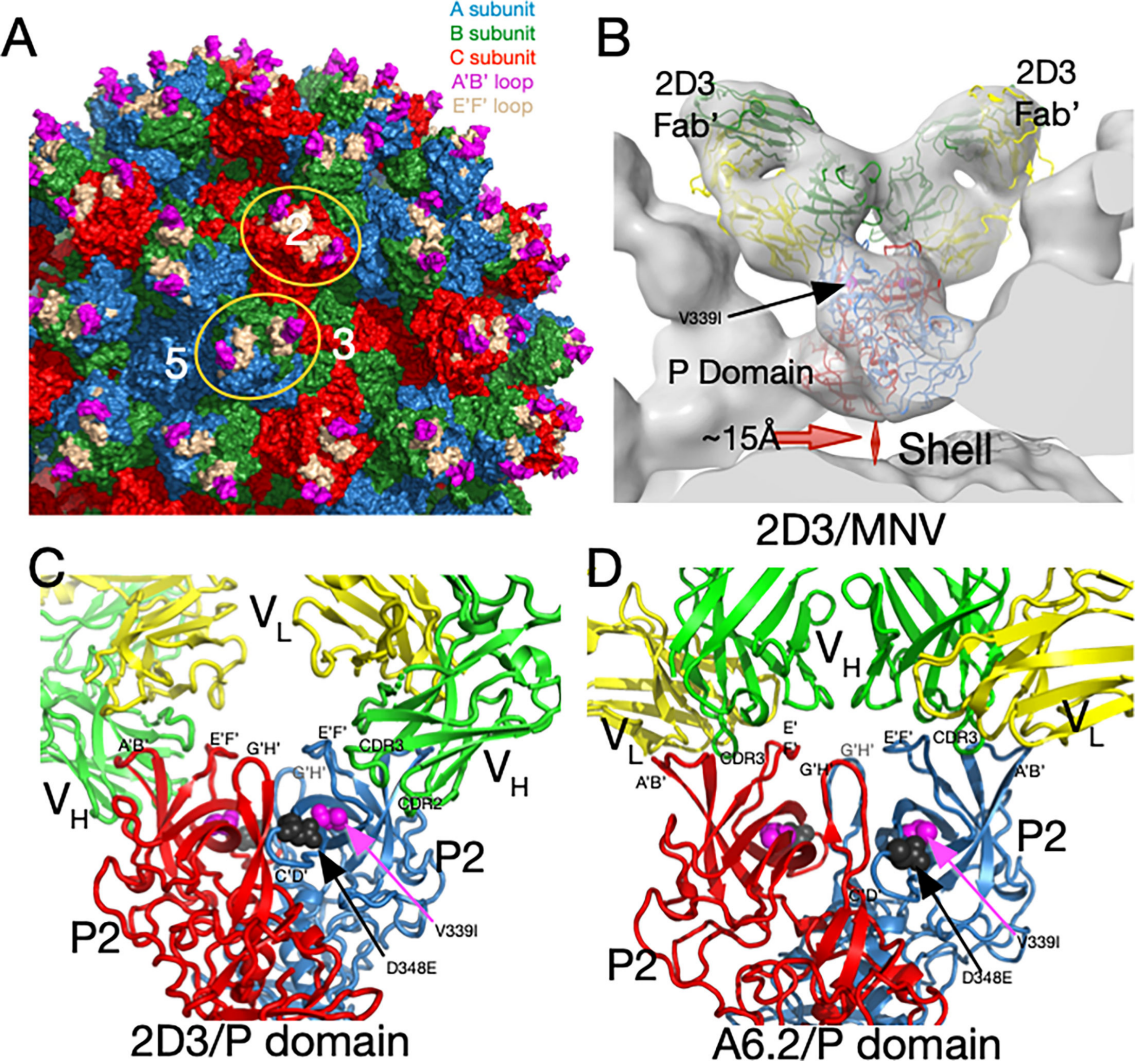
MNV capsid structure and antibody complexes. (**A**) A surface-rendered section of the MNV capsid showing VP1–3 colored blue, green, and red, respectively. A/B subunits form dimers around the fivefold axes, while the adjacent C/C subunits form dimers at the icosahedral twofold axes. Also noted is the location of a fivefold, threefold, and twofold axis. At the tip of the P domain, the A’B’ and E’F’ antigenic loops are colored mauve and tan, respectively. Example A/B and C/C P domain dimers are highlighted by orange ellipses. (**B**) Previous cryo-EM structure (13) of the 2D3 Fab’/MNV complex and the associated pseudo-atomic model (**C**). 2D3 binds to the outermost tip of the P domain and makes nearly identical contacts as the antibody A6.2 (panel D) (13). Residue V339I is highlighted in mauve and D348E in black. In panels C and D, the two P domains are colored red and blue, and the antibody heavy and light chains are colored green and yellow, respectively. The model from this ~8Å map only approximates the antibody/MNV complex but is sufficient to show that the 2D3 epitope lies between the A’B/E’F’ loops. (**D**) Shown here is the 3Å cryo-EM structure of the A6.2 Fab/MNV P domain complex. Note that the Fabs in both antibody complexes have nearly the same P domain contacts, even though the escape mutations to 2D3 are distal to the bound antibodies and A6.2 escape mutants.

The T=3 icosahedral calicivirus capsid is composed of 180 copies of VP1 with a molecular weight of ~58 kDa, which is divided into the N-terminus (N), the shell (S) and the C-terminal protruding (P) domain (1–5) (Fig. 1). The S domain forms a shell around the viral RNA genome, while the P domains form protrusions emanating from the shell comprising A/B and C/C dimers. The P domain is further subdivided into P1 and P2 subdomains, with the latter containing the binding sites for cellular receptors (5–9) and neutralizing antibodies (10–13). For reasons that have not been entirely clear, gut immunity to both human (14) and murine (15, 16) noroviruses quickly deteriorates, and the host can be reinfected by the same strain just months after recovery.

Murine norovirus (MNV, genotype GV.1) is a powerful surrogate for human noroviruses (HNoVs) since it can be grown to high titers in the cell culture, has a reverse genetic system available, and mice serve as the natural host as well as a convenient animal model. The latter is crucial since research on HNoV will always be limited by the fact that mutants/variants cannot be tested *in vivo*. Furthermore, MNV is appropriate as a HNoV surrogate since recent studies on HNoV capsids have recapitulated some of our structural work on MNV (e.g., 17, 18), and our recent publication (19) showed that the histopathological changes in the mouse MNV model mirror the limited findings in human studies (20–23).

In the original MNV-1 P domain X-ray structure (12), the loops at the tip of the P domain (Fig. 1A) adopted two different conformations; the A’B’ and E’F’ loops were either splayed apart (open) or tightly associated (closed). From our ~ 8Å cryo-EM structures of several Fab/MNV-1 (e.g., Fig. 1B) complexes (4, 13, 24–26) and the X-ray crystal structure of one of the Fabs (25), we suggested that the antibodies preferred binding to the “open” loop conformation (Fig. 1B through D). This was confirmed with our structure of the soluble form of the P domain complexed with the Fab of the neutralizing monoclonal antibody A6.2 to 3.2Å (Fig. 1) (27). As we had predicted, the H chain CDR3 loop “unfurls” to reach down to the hydrophobic cleft between the A’B’/E’F’ loops in the “open” conformation but would not recognize the P domains in the “closed” loop conformation.

**Fig 2:**
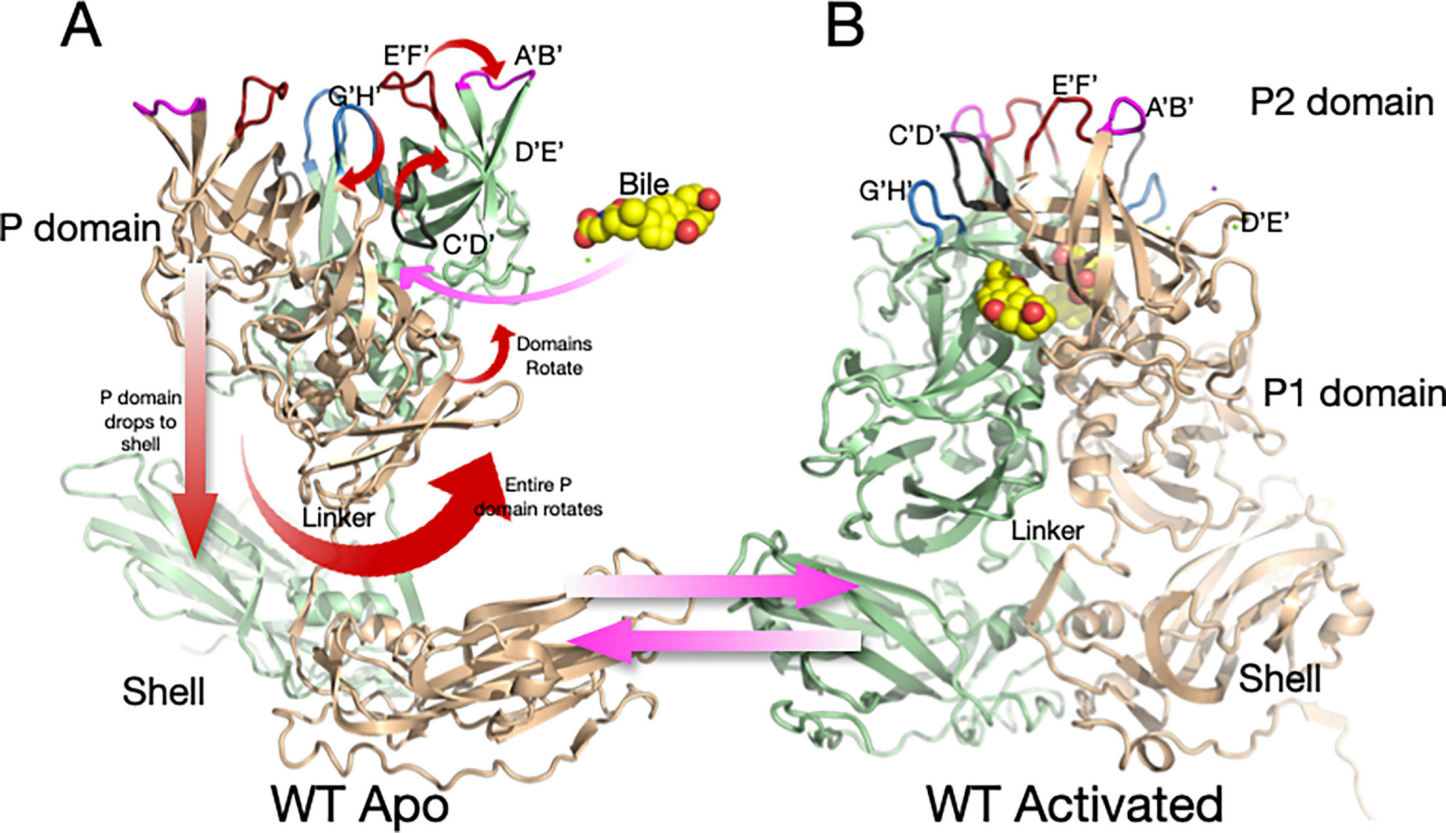
Shown here is the structure of wt MNV-1 in PBS at pH 7.4 (**A**) and the structural changes that occur during activation (**B**). In PBS, the P domain floats above the shell and is highly flexible. The addition of metal ions (i.e., Mg^2+^ or Ca^2+^), bile salts, or acidic buffers causes motions noted by the red arrows, resulting in the structure shown in B.

We have shown that P domains are flexible and display markedly different conformations depending on environmental conditions. In PBS buffer, the P domains of several caliciviruses (MNV, rabbit hemorrhagic disease virus [RHDV], and human norovirus genogroup II [HNoV GII]) are highly mobile and “float” ~15Å above the surface of the shell (4, 12, 24–26), representing the expanded (apo) form (Fig. 2A). Since this structural feature is conserved across calicivirus genotypes and genera (24, 27, 28), we suggested that it likely has an important biological function.

We recently showed that the “apo” (previously described as “raised”) structure markedly changes with the addition of the bile acids (e.g., GCDCA) (5), metal ions (29), or low pH (30) (Fig. 2B). These changes enhance P domain binding to the receptor CD300lf (8) while blocking the binding of antibodies (31). All these activators cause the same conformational changes (29–32). In the apo form of the capsid, the C’D’ loop points down toward the capsid surface and covers the entrance to the bile binding pocket. This loop orientation allows the A’B’ and E’F’ loops to splay apart into the open conformation. In the presence of bile, low pH, or metals, the C’D’ loop moves up and holds the A’B’/E’F’ loops in the closed conformation (5). We hypothesize that the G’H’ loop plays a pivotal role in pH- and metal-mediated activation. The G’H’ and C’D’ loops essentially swap spaces at the tip of the P domain; when one loop is up, the other is necessarily down. At neutral pH, a cluster of acidic groups on the G’H’ loop (D440, D443, and E447) is charged and forces the G’H’ loop into a more vertical (radially outward) orientation, allowing the C’D’ loop to fold over the bile binding pocket. At acidic pH or in the presence of metal ions, the charges are neutralized, and the G’H’ loop folds down into a compact structure, causing the C’D’ loop to lift and push the A’B’/E’F’ loops into the closed conformation that blocks antibody binding while enhancing receptor binding between the A’B’ and D’E’ loops (8). We also demonstrated that all these activators (i.e., bile acids, metal ions, and low pH) cause the P domain dimers to rotate about each other (5, 30–32) (Fig. 2). This rotation changes the surface of the P domain base to complement the shell surface and causes the contraction of the P domain onto the shell (previously described as “resting”). For simplicity, we here call the structure in PBS alone “apo” (with open A’B’/E’F’ loops and a floating P domain), while the structure in the presence of metal ions, bile salts, or low pH (with a closed A’B’/E’F’ and contracted P domain) is the “activated” state. It should be noted that while some previous crystal structures of several caliciviruses were in the contracted state (2, 33, 34), our work suggests this may be due, at least in part, to the low pH (i.e., pH 4.7–5.6) of the crystallization conditions.

To further explore the dominant epitopes and mechanisms of antibody neutralization, additional antibodies against MNV-1 were produced (13, 26). These new monoclonal antibodies, 2D3 and 4F9, broadly neutralize all MNV strains tested (13). Furthermore, a panel of A’B’/E’F’ loop escape mutants to monoclonal antibody A6.2 failed to block 2D3 neutralization (26). The only escape mutants to 2D3 and 4F9 were V339I and D348E, respectively (Fig. 1D through F) (13). Since these residues lie on the C’D’ loop and are ~20Å away from the binding site of A6.2 (26), it was presumed that 2D3 would bind well away from the A6.2 epitope. However, and rather surprisingly, 2D3 binds to the same general location as A6.2 (13). We therefore concluded that these escape mutations act in an “allosteric” manner by indirectly causing conformational changes in the epitope. Herein, we set out to understand how these escape mutations can affect antibody neutralization at such a distance.

The goal of our studies is to elucidate the V339I and D348E escape mechanisms and why the 2D3 and 4F9 antibodies broadly neutralize all strains of MNV (26). As reviewed above, at neutral pH and in the absence of metal ions or bile salts (PBS buffer), MNV adopts the “apo” structure where the P domain is ~15Å off the shell surface, and the A’B’/E’F’ loops are in the open conformation (Fig. 2A). However, we show here that the allosteric escape mutants, V339I and D348E, are in the “activated” state in PBS where the P domain is tightly associated with the shell and the A’B’/E’F’ loops are closed and thus sterically block antibodies from binding (Fig. 2B). These loops are less ordered than when metal ions or bile salts are added, but the escape mutants nearly fully activate the virus without stimuli. Further, while these escape mutants arose using the 2D3 antibody, they also block neutralization by the A6.2 antibody. The reason we did not previously isolate V339I and D348E escapes in the presence of A6.2 is likely because the other mutations in the E’F’ loop were sufficient to block binding while being more fit than the V339I and D348E mutants. As shown in Fig. 2, this transition between apo and activated structures involves many steps, and it has been unclear as to the order or importance of each. To better understand this transformation and its regulation, all-atom molecular dynamics simulations were performed starting from the crystal structure of the apo form (12) with and without the V339I mutation. This starting structure presents both the “open” and “closed” conformations of the A’B’/E’F’ loops in the dimer, with the C’D’ loop in the down position. While the A’B’/E’F’/D’E’ loops were highly mobile during the simulations, the C’D’ loop remained intransigent, suggesting that its movement is far slower than that of the other, less constrained, loops at the tip. The V339I P1 domain was far less mobile than the P2 domain, but the two domains in the dimer rotated about each other, akin to what happens during activation (29–31, 35). Finally, the A’B’/E’F’ loops were more mobile in the V339I than wild-type (wt), suggesting that the mutation increases the dynamic nature of the outer loops, and perhaps this facilitates the transition to the “closed” state in the absence of activators.

In summary, this highly mobile virus not only leverages conditions in the gut lumen to block antibody binding but can also trigger these changes via allosteric escape mutations. These results may offer a possible explanation for the transient immune response to norovirus infection and strongly imply that vaccines can be greatly improved by using the activated structure found in gut conditions as antigens.

## RESULTS

### Antibody neutralization

We have previously isolated and characterized three neutralizing monoclonal antibodies (mAb) to MNV-1 called A6.2, 2D3, and 4F9 (4, 12, 13, 25, 26, 30, 31) with different neutralization properties and non-overlapping escape mutations. The first mAb studied, A6.2 (36), is an IgG antibody that binds between the A’B’/E’F’ loops (Fig. 1D) (31). A naturally occurring escape mutation was found in the E’F’ loop at residue L386 (37), and subsequent studies identified additional escape mutations in the E’F’ loop at amino acid positions V378, A382, and D385 (25). All these residues were either in contact with bound A6.2 or immediately adjacent. Subsequently isolated antibodies, 2D3 and 4F9, are both IgA antibodies and neutralized the A6.2 E’F’ loop escape mutants (26). The only naturally occurring escape mutations to these antibodies were in the G’H’ loop (D348E and V339I) that do not contact the bound antibody (13).

**Fig 3:**
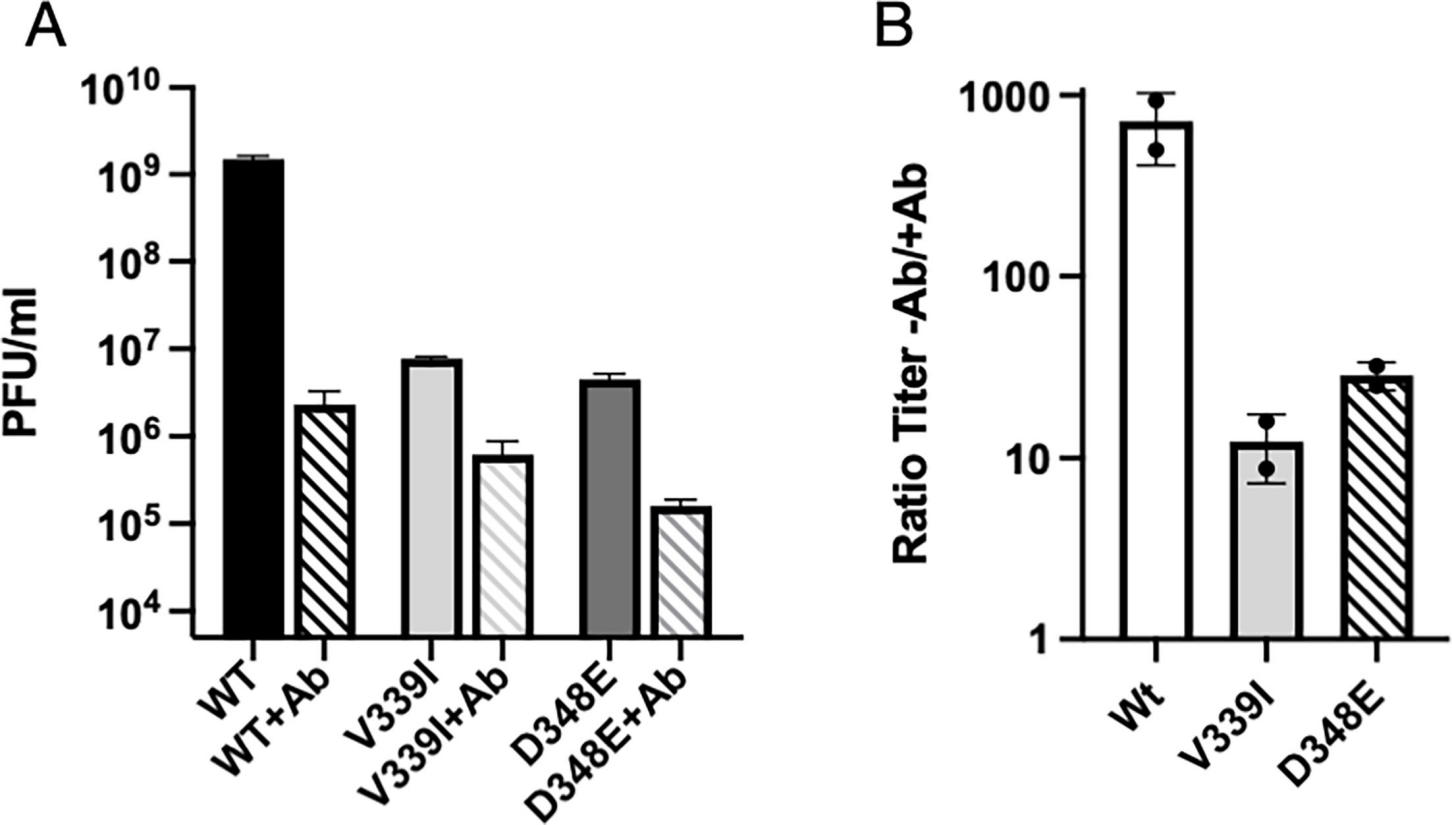
A6.2 neutralization of wt and the V339I and D348E escape mutants. (**A**) The titer of the three viruses in the presence and absence of 50 µg/mL monoclonal antibody A6.2. (**B**) Since the viruses all had different initial titers, the ordinate axis in this graph shows the titer in the absence of the antibody divided by the titer in the presence of the antibody. As shown here, wt was neutralized by more than three logs, while both mutants were only neutralized by one log.

While we previously showed that 4F9 and 2D3 neutralize viruses with the A6.2 escape mutations on the E’F’ loop (26), we did not test whether mutant viruses with amino acid changes D348E and V339I are neutralized by A6.2. As shown in Fig. 3, both mutants are very effective at escaping neutralization by all antibodies. Therefore, whatever conformational changes are induced by the V339I mutation, it disrupts the epitopes for all three antibodies. It should be noted, as with all these types of plaque assays, that the antibody concentration is orders of magnitude greater than the virus under all conditions tested. Furthermore, all three antibodies were successfully used during large-scale virus production to prevent the emergence of wild-type revertants. It is interesting and relevant to note that while A6.2 recognized the MNV capsid protein in Western blots (25), neither 4F9 nor 2D3 can (26). It may be due to the fact that A6.2 recognizes the outermost portions of the A’B’ and/or the E’F’ loops that are better reconstituted in a Western blot, while 2D3 and 4F9 are necessarily interacting with more of the 3D structure at the tip. This suggests that while all three antibodies bind to approximately the same region of the P domain (13), they differ in which residues are critical for binding with, perhaps, A6.2, mostly recognizing the linear epitope on one or the other loop. As an example of this phenomenon, none of the highly neutralizing antibodies to human rhinovirus 14 (HRV14) react in a Western blot, and the structure of the antibody/virus complexes shows that antibodies extensively interact with ~1,000 Å^2^ of the surface (38–40).

### Structure of V339I +/- activators

**Fig 4:**
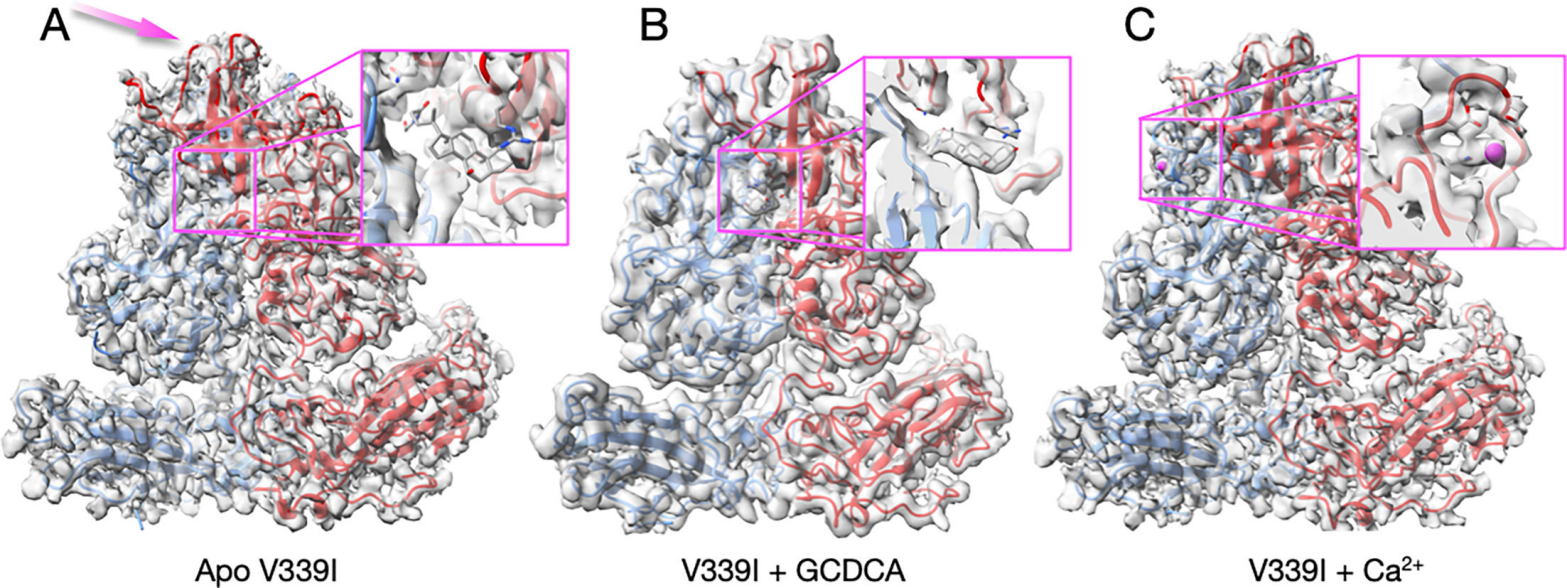
Cryo-EM structures of the V339I mutant virus apo (**A**), complexed with GCDCA (**B**), or calcium (**C**). (**A**) In contrast to the apo wt virus (Fig. 2A), the P domain is contracted onto the shell even in the absence of bile salts or metal ions. The one notable difference is that the outermost loops in apo V339I (panel A, mauve arrow) are less ordered than in the presence of either GCDCA or Ca^2+^. The inset figure shows where GCDCA would bind if it were present. The lack of density demonstrates this was indeed an apo sample. (**B**) The V339I+GCDCA structure is also in the activated state, and the inset shows clear density for the bound bile salt. The outermost loops are better ordered than in the apo form. (**C**) The density and structure of V339I+Ca^2+^. As with the GCDCA complex, the outer loops are better ordered. The inset shows calcium bound to the G’H’ loop and was not observed in either the bile-bound or apo forms of V339I.

To understand the mechanism of antibody escape, we performed structural studies of the escape viruses V339I and D348E, starting with V339I. Once the sequence of the purified mutant virus had been verified to contain the V339I mutation, the structures of V339I apo, V339I + 2 mM CaCl_2_, and V339I + 10 mM GCDCA were determined from that same sample. The calculated resolutions for all three cryo-EM reconstructions were ~2.7Å. The most striking finding was that, unlike the apo wt virus in PBS (Fig. 2A), apo V339I was in the activated state with the P domain rotated and lying on the surface of the shell (Fig. 4A). As evidence that this is truly V339I without activating ligands, the inset in Fig. 4A shows the absence of density where GCDCA would be located. The density of the outermost loops (mauve arrow, Fig. 4A) is disordered, and for these regions, the unsharpened reconstructions were used to better place the main chain atoms. Fig. 4B shows the structure of V339I+GCDCA. There is clear density for GCDCA (inset), and the loops at the tip are more ordered than with apo V339I (examples of the loop densities are shown in Fig. S1). Similarly, V339I+Ca^2+^ is in the fully activated state with the P2 domain loops, and bound calcium is clearly defined (Fig. 4C). The inset shows the density of the G’H’ loop with a bound metal calcium ion. Therefore, the V339I mutation is sufficient to convert the virus to the activated state at neutral pH, even in the absence of metals and bile salts, but adding activators enabled the virus to further enter into the activated state by stabilizing the outer loop conformations. We have previously shown that the neutralization of MNV by A6.2, 2D3, and 4F9 is all blocked when the virus is in the activated state (5, 30–32). Therefore, this mutation-driven activation process is a simple answer as to how V339I can allosterically block neutralization with all three antibodies (26) (Fig. 3).

**Fig 5:**
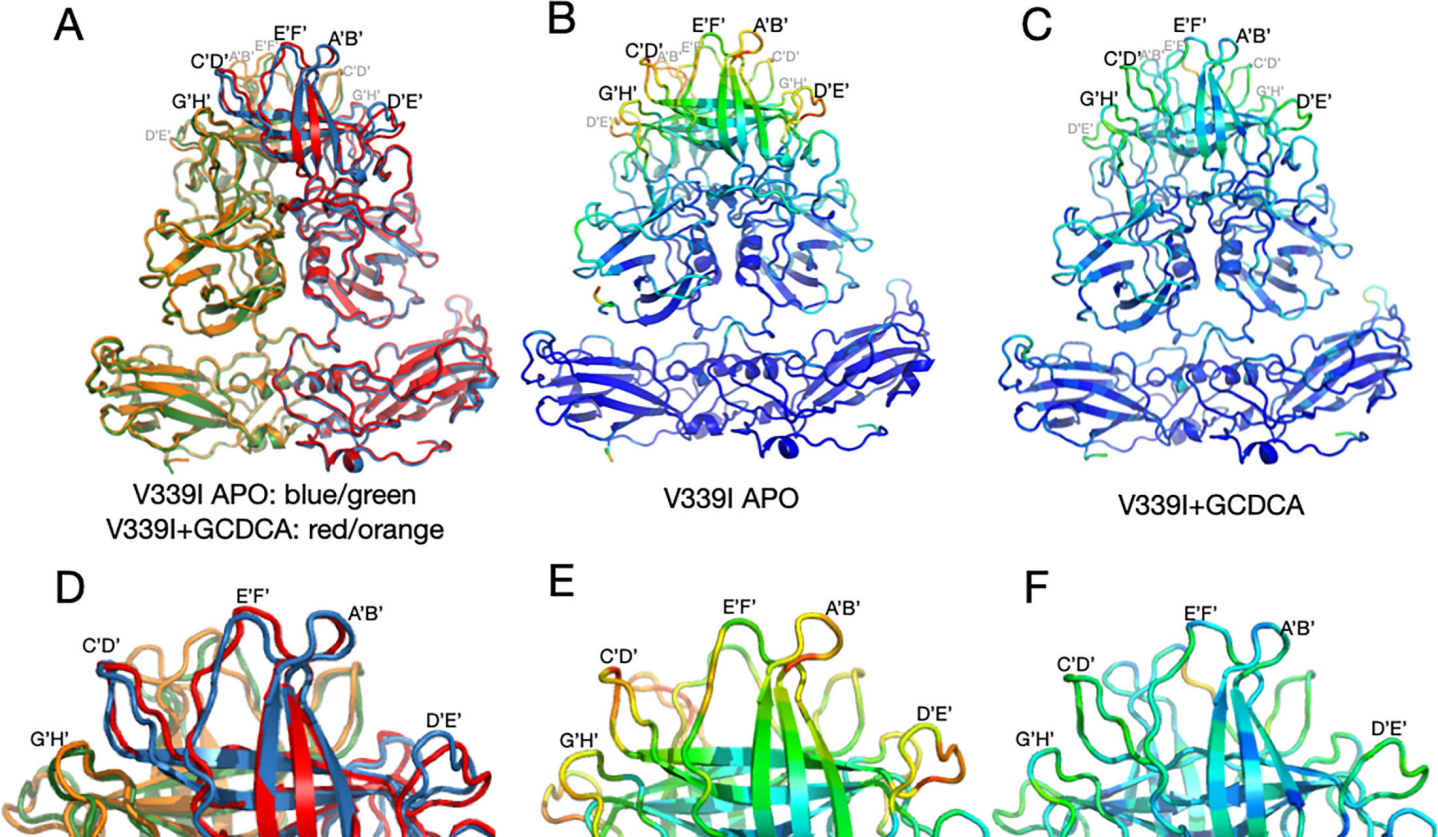
Comparisons of the various V339I structures. (**A**) Overlay of the apo V339I structure (blue/green) onto the V339I+GCDCA structure (red/orange). While the shell and the P1 domains are essentially identical, the structures of the loops at the tip of the P2 domain diverge, with the density of the loops in the apo structure being weaker than in the bile-bound structure. (**B and C**) Shown here is the per-residue CC for the bile-bound (**B**) and apo (**C**) mapped onto the structure as B-factors using the formula 100(1 CC). While shell and P1 domains for both structures match their respective densities very well, the outer loops of the apo structure are not well-defined (red and yellow colors). Panels D, E, and F are enlarged images of the loops in A, B, and C, respectively. Note that bile stabilizes the conformations of the outer loops as per the shift from red/yellow to blue/green.

Comparisons of the structures of V339I +/– activators are shown in Fig. 5. It should be noted that this is the first time we could compare MNV virus structures in the same conformation +/– activators. Panel A shows an overlay of V339I apo (blue and green) onto V339I+GCDCA (red and orange). The positions of the P domains are essentially identical and are in the activated state, resting on the shell surface. The overall RMSD of the alignment between these two structures is ~0.4Å, well within the margin of error at this resolution. The only apparent differences between the two structures are in the outermost loops that are more than likely due to inherent flexibility. To highlight the regions of highest disorder, the ribbon diagrams in Fig. 5, panels B and C are colored red to blue as the correlation coefficients (CC) of the residues extend from 0 to 1.0. The correlation coefficients are calculated during real space refinement in PHENIX and represent the agreement between the refined model and real space density. These outermost loops become more ordered with the addition of GCDCA or Ca^2+^ (the color shifts from reds/yellows to blues/greens), which is likely due to the stabilized positions of the G’H’/C’D’ loops (insets in Fig. 5A through C).

**Fig 6:**
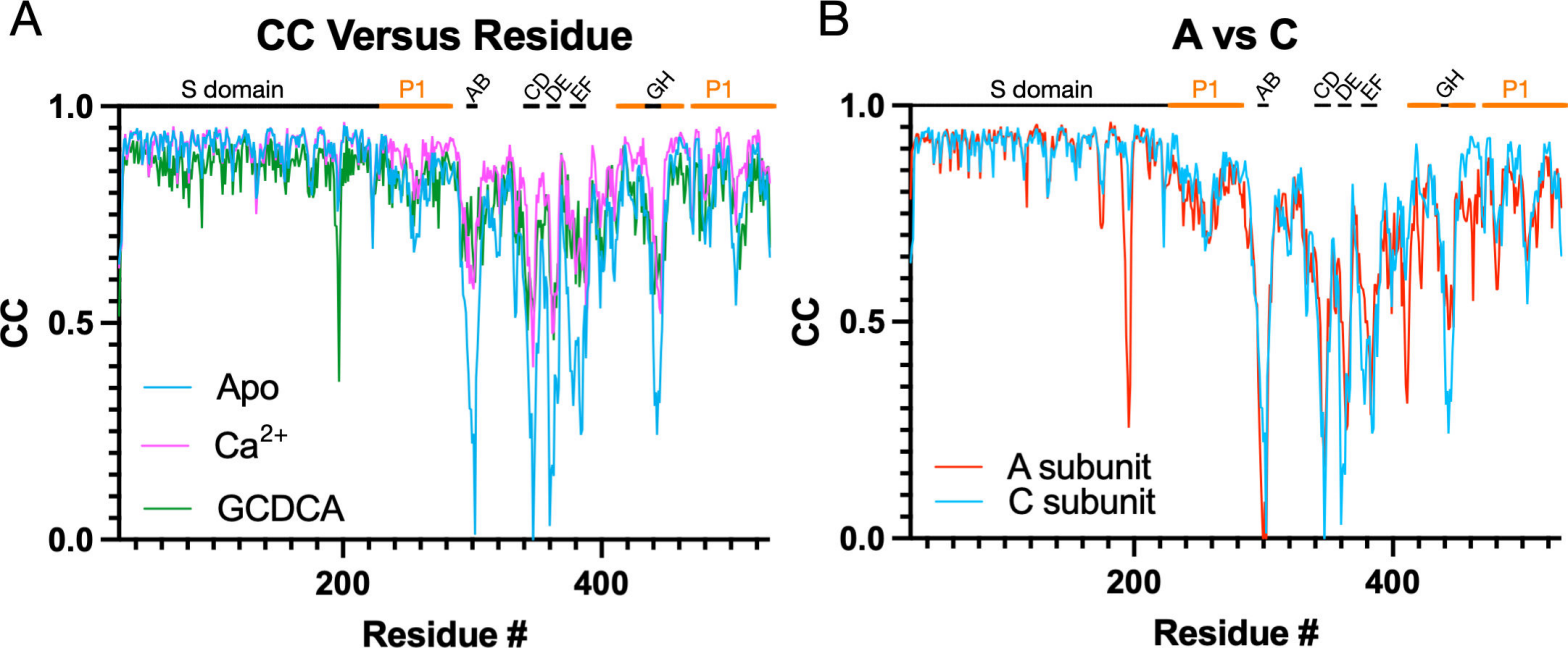
Comparisons of the per-residue correlation coefficients among the three structures. (**A**) Shown here are the per-residue CC for the C subunits in the presence and absence of bile or calcium ions. The locations of the outermost loops are noted at the top of the graph, and the P1 domain is noted by the orange bars. Although the apo form is in the activated state, the loops are significantly less ordered than when bile or calcium is present. (**B**) Shown here are the per-residue CC of the A versus C subunits in the apo V330I structure. While both structures have highly mobile loops, the A subunit appears less ordered in several loops and from residue 400 onward.

These changes in loop flexibility are further shown in Fig. 6. In panel 6A, the per-residue CC for the C subunits is plotted for the three structures. The overall CC for the P domains alone was 0.69, 0.82, and 0.76 for the apo, +Ca^2+^, and +GCDCA, respectively. The S subunits for all three structures had the highest CC and therefore were the least mobile portions of the capsid. Slightly more mobile than the S domains were the P1 domains (denoted by the orange bars above the graphs). The P2 domain was the most flexible part of the P domain, with the outermost loops having extremely weak density in places (see Fig. S1). The addition of activators (i.e., GCDCA or CaCl2+) decreased the mobility of this P2 domain as per the improved CC. Therefore, while the V339I mutation alone is sufficient to push the P domain nearly into the fully activated conformation, the addition of these activators finishes the activation process and stabilizes these key loops. We had previously noted that there are also differences between the A/B and C/C dimers (5). These dimers differ in their interactions with the shell and with other dimers. The overall CC for the V339I apo P domains of the A and C subunits are 0.69 and 0.67, respectively. While this is a relatively small difference, the densities for the loops and the carboxyl end of the protein appear to be consistently weaker (Fig. 6B). This suggests that the context of the P domain within the icosahedron may also influence the mobility of these loops.

**Fig 7:**
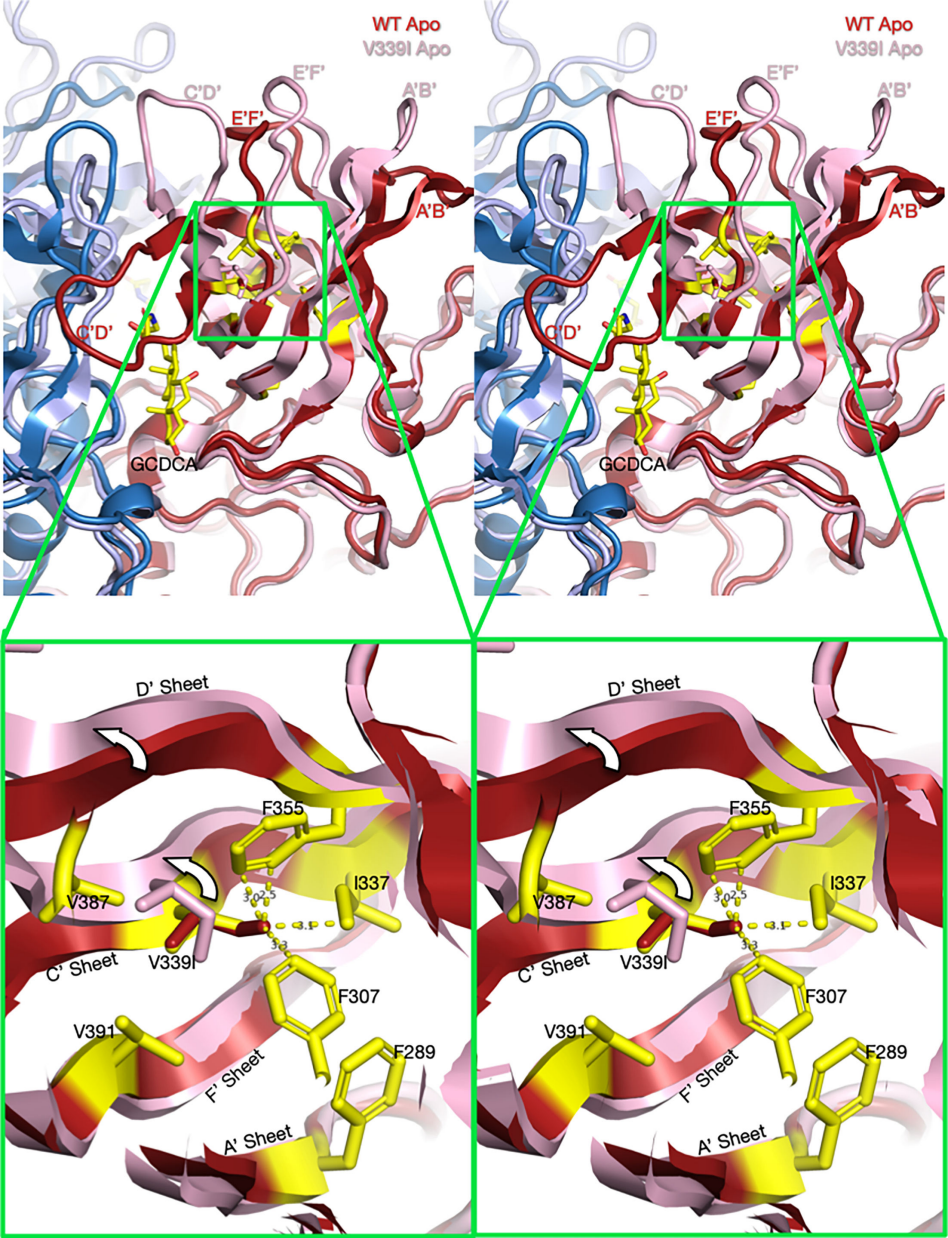
Stereo diagrams of the area surrounding the V339I mutation and possible mechanism for activation (Videos S7 and S8). The top stereo pair shows a wt apo P domain dimer (4, 24) in deep red and blue. Overlaid on that structure is the apo V339I structure in pink and pale blue. To help define the location and orientation of the figure, the bound GCDCA from the V339I/GCDCA structure has been added. Note that V339I lies at the N-terminal side of the C’D’ loop that moves up drastically in the wt virus at low pH or when metal ions or bile salts are added. In the magnified view, V339 was replaced with an Ile (red) in the apo wt structure. As noted in this figure, the extra methyl group of the isoleucine would be too close to F335, I337, and F307 in this tightly packed hydrophobic pocket. However, in the actual apo V339I structure, the C’ and D’ β-strands move up, away from the core, and make room for the larger isoleucine side chain.

Using the structures of V339I in the activated state and apo wt that is recognized by antibodies (31), we can hypothesize how this allosteric escape mutant causes activation of MNV. Fig. 7 and Videos S7 and S8 show the apo structure of wt MNV (red and blue) aligned with the activated structure of the V339I mutant (pink and light blue). Highlighted in yellow are the side chains of the apo wt structure in the immediate vicinity. Overlaid onto the V339 sidechain is a simple substitution with an isoleucine (red). In the apo wt structure, the environment of V339 is markedly hydrophobic and tightly packed. There is simply not enough space in this cavity to accommodate the extra methyl group of Ile that would clash with I337, F307, and F355. We propose that the mutation causes a shift up in the C’ and D’ strands that results in the upward movement of the C’D’ loop. Therefore, this overcrowding mutation is sufficient to push the conformation of the C’D’ loop into the “up” position, leading to the cascade of other conformational changes that activate the virus (Fig. 2).

### Structure of apo D348E

**Fig 8:**
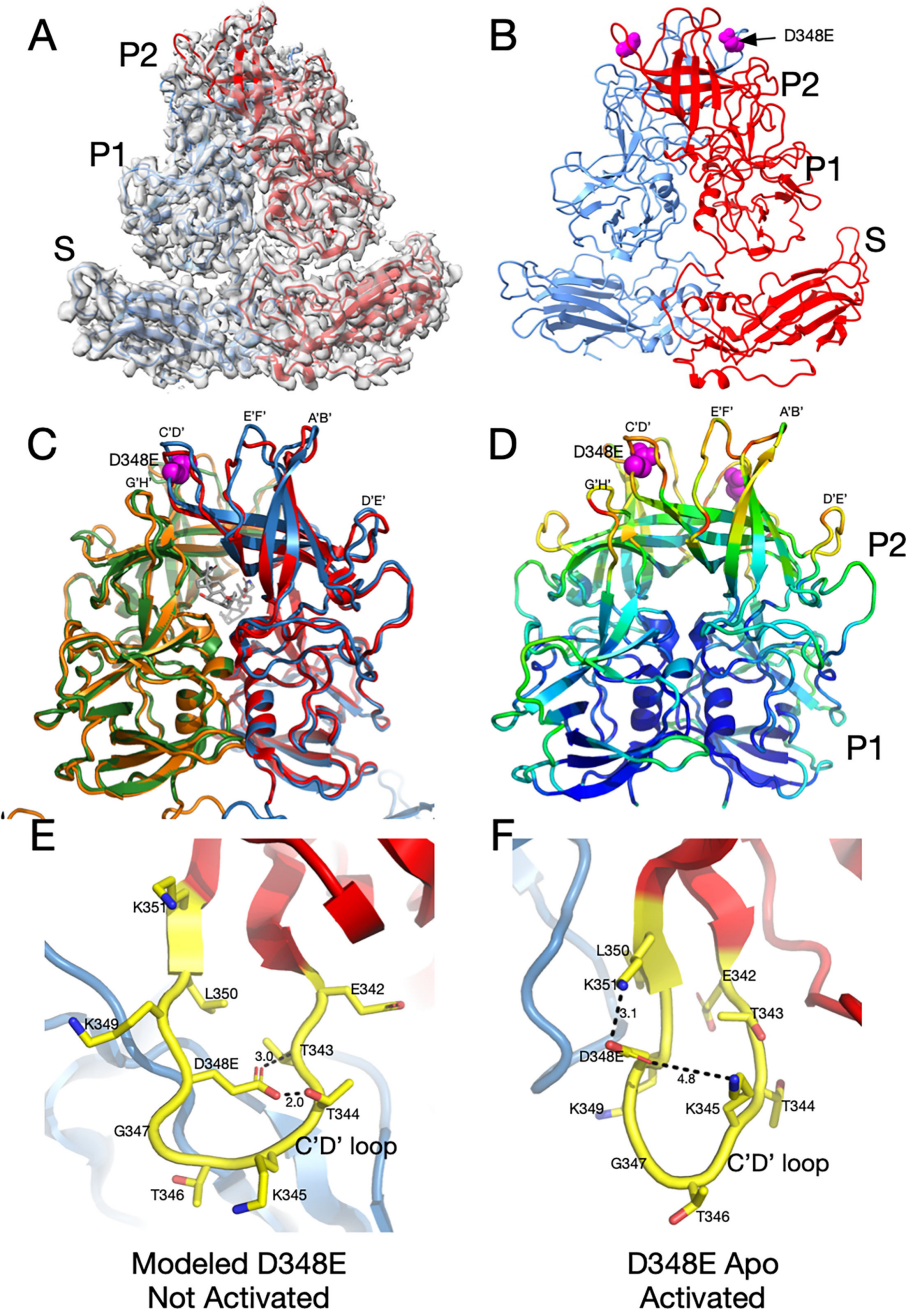
Structure of the D348E allosteric escape mutant and possible mechanism of activation (Videos S9 and S10). (**A**) The 3Å EM density of apo D348E and the corresponding model. The two subunits in the dimer are colored red and blue. Note that, as with V339I, the P domain of apo D348E is resting on the shell in the activated conformation. (**B**) Shown here is the apo D348E structure alone with the location of the mutation site denoted by the mauve spheres. (**C**) An overlay of the apo D348E structure (blue/green) with the cryo-EM structure of wild-type with GCDCA bound (red/orange). The location of the D348E mutation is noted by the mauve spheres, and the bound GCDCA (wild-type structure) is represented by the gray model. (**D**) This is the P domain portion of the apo D348E structure colored according to the CC, ranging from blue (1.0) to red (0.0). (**E**) Shown here is a modeled structure of D348E in the unactivated conformation (Video S9). As with V339I, since the D348E mutation forces the virus into the activated conformation, the mutation needed to be modeled into the wt apo conformation. D348 was mutated in COOT and assigned the most likely rotamer position. The distances in this hypothetical model are only shown for reference. The larger sidechain collides with the T343 and T344, and therefore the mutant C’D’ loop cannot adopt this conformation. (**F**) Shown here is the actual structure of Apo D348E that is in the activated conformation where the C’D’ loop is lifted away from the shell (Video S10). This places D348E out of the plane of the loop, and it extends upward where there is sufficient space and places D348E into a favorable electrostatic environment adjacent to K351 and K345.

To understand whether V339I and D348E escape mutants use the same mechanism of escape, we next determined the structure of the D348E virus. D348E is on the exposed end of the C’D’ loop, far removed from the antibody binding site (Fig. 1C and D). Since D348E can escape neutralization by all three monoclonal antibodies, it was expected that, like V339I, the apo structure would also be in the activated conformation in PBS. As with the V339I mutant, the sample used for structural studies was sequenced immediately prior to cryo-EM data collection and verified to contain the mutation. Fig. 8A and B show that, indeed, apo D348E is in the activated state where the P domain is resting on the shell. The loops in apo D348E are in the same conformation as the wt under activating conditions (Fig. 8C), but far more disordered (Fig. 8D). Therefore, the D348E allosteric escape mutation activates MNV in a similar manner as V339I (see Videos S9 and S10).

We proposed above that the V339I mutation pushes the P domain to the activated state by being too large for the space occupied by the C’D’ strands and causing movement in the C’D’ loop and subsequent effects throughout the P domain. Since the P domain in the D348E mutant is in the activated conformation without any gut triggers, we can only model what may happen when the aspartate is mutated to glutamate in the apo conformation of the wt virus (Fig. 8E). The side chain of D348 points toward the N-terminal side of the C’D’ loop. When the aspartate is replaced with a glutamate in the modeled structure, it is too large and collides with T343 and T344. However, in the activated conformation of apo D348E, the D348E side chain rotates up, and there is more than enough space to accommodate the larger side chain. In addition, this orientation places the acidic side chain in an electrostatically favorable location, adjacent to K351 and K345. Therefore, D348E could cause a push/pull structural change where the clashes caused by the mutation push the C’D’ loop out of the wt apo conformation, while simultaneously being pulled toward the activated state conformation because of favorable electrostatic interactions with adjacent lysine residues. Movement in the C’D’ loop is then anticipated to cause a domino effect in the P domain, leading to closed A’B’ and E’F’ loops at the tip of the P domain, a structural conformation which is resistant to neutralization by all three mAbs.

### Molecular dynamics simulations

What we have shown so far is only the static apo and activated structures of MNV, leaving much unknown about the transitional states that conform one structure into the other. To consider this conformational transition and how V339I might affect it, a series of molecular dynamics simulations were performed. In these two simulations, we used unbiased all-atom simulations to study the effect of the V339I mutation on the conformational stability of the P domain of MNV and to better understand how this mutation hinders antibody binding. The simulations started from the crystal structure of the apo P domain (12) that has chain A in the open conformation, chain B in the closed conformation, and the C’D’ loop pointing down away from the top. Interestingly, this crystal structure suggests that, with the C’D’ loop pointing down, the A’B’/E’F’ loops can adopt both conformations. With the two subunits in “opposite” conformations, the simulations could show changes in the apo conformation of the V339I mutant using the same model and in the same computation.

The radius of gyration (Rg) is a measurement of the size and compactness of the structure, and values were calculated using all non-hydrogen atoms. As shown in Fig. S2, both wt and V339I remain stable during the simulations and are well-converged. However, the V339I mutant possibly exhibited slightly more fluctuations as per greater deviations from the mean radius.

The conformational changes were sampled over 900 ns, after 5 ns of equilibration. For the root-mean-square deviation (RMSD) calculations, each snapshot was first aligned to the first frame after equilibration using the non-hydrogen atoms of the backbone of the two subdomains, P1 and P2. The RMSD reflects the room temperature flexibility of the MNV and, specifically, of the P2 domain where the A’B’ and E’F’ loops fluctuate. As shown in Fig. S3, there is a marked difference between the P1 and P2 domains, where the P2 domain exhibited far more conformational movement than the P1. The P1 domain structure converged quickly and exhibited fluctuations of ~1.3Å. In contrast, the root-mean-square deviation of the P2 domain varied between 2 and 3Å, with occasional, short-lived sampling of conformations with RMSD values between 3 and 3.5Å. The system was apparently converged after 200 ns with respect to that reference frame but not to one single state. These findings that the inhomogeneous fluctuations in the P2 domain are much larger than the P1 domain recapitulate our experimental data (e.g., Fig. 6 and references 13, 35).

**Fig 9:**
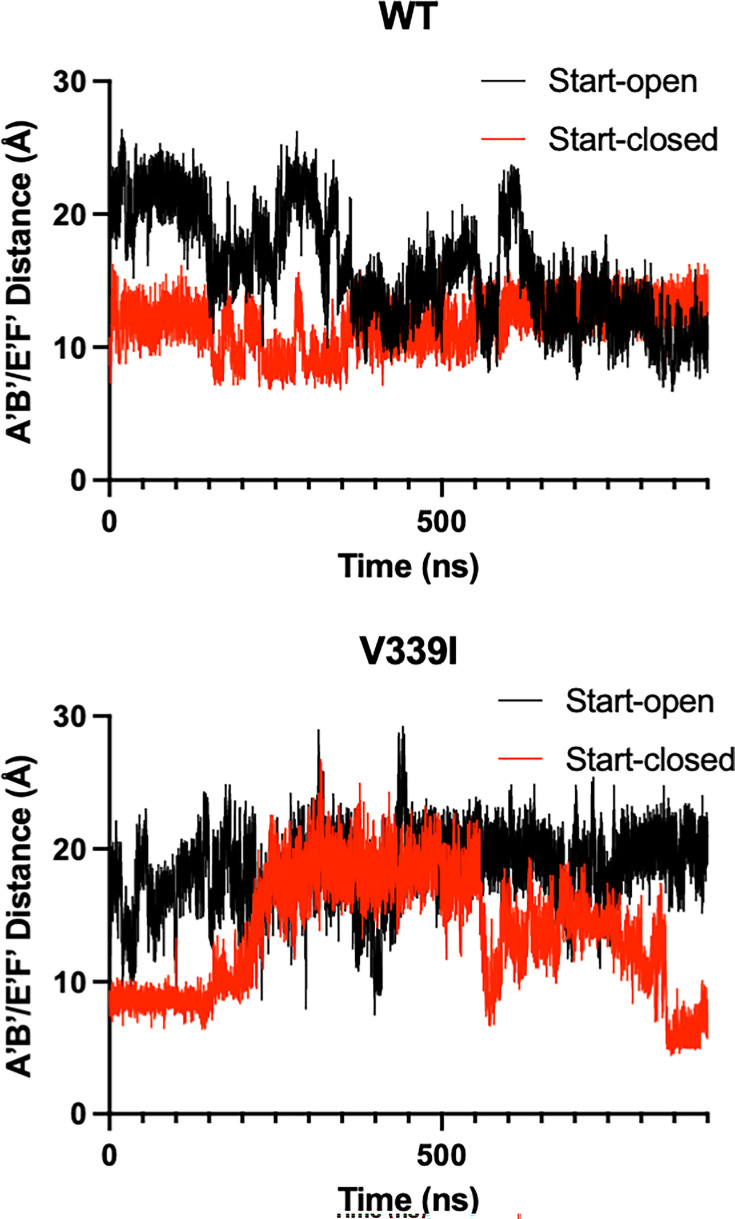
Movement in the A’B’/E’F’ loops during molecular dynamic simulations. The simulations were started with the apo crystal structure of the P domain where the A subunit (black lines) is in the “open” position and the B subunit (red lines) is in the “closed” position with an average distance of ~20Å and ~10Å, respectively. With the wt structure, the two subunits converged to approximately the closed conformation (Video S5). In contrast with the V339I structure (wt with V339 replaced with an isoleucine), the V339I A subunit (open) does not converge to the closed conformation but does briefly sample an approximate conformation during the calculation (Video S6). V339I appears to destabilize the conformation of the A’B’/E’F’ loops.

The RMSD observed in Fig. S3 is mainly a consequence of fluctuations of the two key loops of the P2 domain: the A’B’ (residues 289–296) and E’F’ (residues 380–384). In these simulations, when the A’B’/E’F’ loops are in the “closed” conformation, the distance between them is ~10Å with the A and B chains started in the open and closed positions, respectively, for these simulations (Fig. 9). There is an apparent difference in the A’B’/E’F’ loop flexibility between the apo and the V339I mutant, despite the mutations being distal to both loops. With the wt P domain, both the A (open) and B (closed) chains took ~600 ns to move to a state with both loops in the closed conformation (Video S5). In contrast, the V339I A (open) conformation did not converge to a state with the closed conformation, but the closed conformations were briefly sampled during the calculation (Video S6). Specifically, at ~400 ns, A and B briefly switched conformations but then returned to their original states, suggesting a facile equilibrium between the states. Interestingly, unlike wt, V339I does not form a stable structure where both A and B have the same open/closed conformation. For both wt and V339I, the C’D’ loop did not move to the “up” position, and in fact, that region was not sampled. Therefore, the C’D’ loop movement takes a much longer time than by these simulations, indicating a barrier between states or different solution conditions needed for that movement to be observed.

### P1 movement

The radius of gyration and RMSD (Fig. S2 and S3 illustrate that the P1 domains are stable and fluctuate about a mean structure during the simulation. For each chain, the P1 domains maintain conformational similarity to the original apo starting structure. However, there is a rotation of the A/B P1 domains about each other (Fig. S4). To quantify this motion, the angle between the α1 helices was measured. For each chain, a vector is defined by the Cα atoms of residues 457, 459, 461, and 463. The angle between those two vectors was then determined as shown in Fig. S4. The angle between the two helices fluctuates between 110° and 140° degrees with a mean value, shown by the red circle, of 125° (wt) versus 123° (V339I), with a standard deviation of 5°. A small rotation of chain B relative to chain A was similarly observed experimentally (Fig. 2 (29–31, 35)). Like the radius of gyration calculations (Fig. S2), the angle between the P1 domains of V339I may be slightly more fluid than that of the wt (as per the greater deviations from the average angle), suggesting less conformational stability and more motion.

### Conclusions

The studies described herein were undertaken to understand how the V339I and D348E mutations block antibody neutralization, even though they are ~20Å from the epitope. Our previous work showed that mAbs 2D3 and 4F9 neutralized all the A6.2 escape mutants (26). Together with the fact that the escape mutants to the 2D3/4F9 antibodies were distal to the A6.2 epitope, it seemed logical at the time to assume that 2D3/4F9 bound in a different location than A6.2. However, this assumption was dispelled with the cryo-EM structure of the 2D3 Fab’/MNV complex (13). While the orientation of the bound 2D3 was not identical to that of A6.2, there was extensive overlap of antibody contact surfaces at the top of the P domain, distal to the locations of the escape residues V339I and D348E. Indeed, Fig. 3 clearly shows that these escape mutants block all three antibodies. The fact that those two escape mutations did not arise in the presence of A6.2 suggests that 2D3 and 4F9 make more extensive contact and are harder to thwart. The structures of the V339I and D348E mutants demonstrate they escape all antibody neutralization processes by converting MNV into the activated form without the need for activators. Both appear to accomplish this by crowding regions of the C’D’ loop with larger amino acids to force it out of the apo conformation and into the “up” activated position. Having V339I in the same “activated” conformation in the apo, +Ca^2+^, and +GCDCA forms also allowed us to conclude that the A’B’/E’F’ loops become far less mobile when activators are added.

The molecular dynamics simulations recapitulated many of these observations and finally lent insights into the relative importance of the loop movements, as shown in Fig. 2. In both wt and V339I simulations (Fig. 9), the C’D’ loops were not observed to rotate to the “up” position found in fully activated P domains, as observed in the activated P domain structures (29–32, 35), indicating such a change would take more than a microsecond kinetically. Nevertheless, the A’B’/E’F’ loops were able to sample the open and closed conformations, with V339I appearing to be more fluid in these motions. Therefore, it seems likely that the C’D’ (and G’H’) loop movement controls the activation process by limiting the conformational space for the A’B’/E’F’ loops rather than imposing a structural change. Interestingly, the simulation also showed that the A and B subunits rotate about each other (Fig. 2), which is necessary to facilitate the contraction of the P domains onto the shell. Again here, the V339I mutant appears to be more mobile than wt. Therefore, these studies showed that the A’B’/E’F’ loop movement and P1 domain rotation can occur spontaneously in the wt virus and is accentuated by the V339I mutation. Therefore, the C’D’ loop acts more like a “gate keeper” by limiting the conformational space of the A’B’/E’F’ loops rather than directly imposing a particular conformational change. By being located on the C’D’ loop, the V339I and D348E mutations may decrease the energy required for the full activation process to occur.

This ease of conformational changes might predict the presence of intermediate virus states with mixtures of raised and retracted P domains in the virions. However, no such intermediates were observed in the raw cryo-EM data or 2D classes. This suggests that the rotation and retraction of the P domain onto the shell is a cooperative process. This cooperativity may be due to changes in the interactions between the P domain spikes in the icosahedron. When the P domains are resting on the shell, the A and C subunits make ~260Å^2^ contact in the icosahedron (5). This connects the P domain dimers around the fivefold axes to those at the twofold axes. As the P domains rotate and lift off the surface (apo structure), interactions switch to the P1 domains (24). Furthermore, the rotation within the dimer during activation (19, 29–31) may switch the P1 interactions in the apo form to P2 interactions in the activated form. Therefore, these interactions may be the cooperativity that switches the P domains from one state to the other, with the activators supplying the small input of energy necessary to start the transition. In this way, activation is initiated with the loss of P1 interactions with the pull of P2 interactions and newly formed complementarity with the shell.

Interestingly, our recent work on the MNV strain CR6 represents a case quite the opposite of these allosteric mutants. CR6 is less infectious (19) and requires multiple stimuli for full capsid activation. While V339I and D348E are nearly in the fully activated state without stimuli, CR6 requires both low pH and bile to fully activate (19). At low pH alone, the CR6 P domain has dropped onto the shell but remains markedly disordered. This is greatly improved upon the addition of bile. Therefore, while the P domain overall has only two positions (floating above or collapsed onto the shell), the loops at the tip of the P domain have multiple conformations that become restrained upon activation.

**Fig 10:**
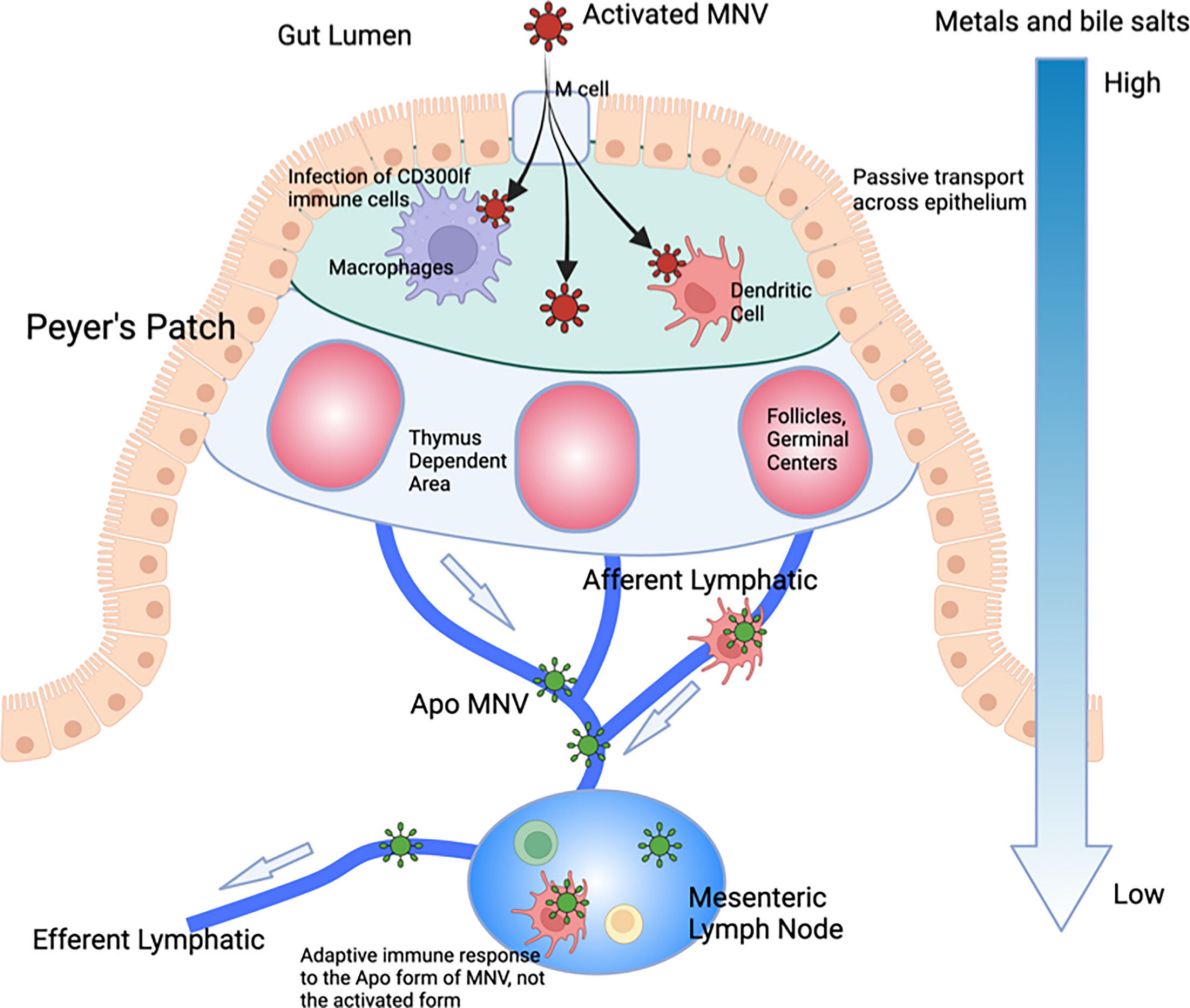
Possible function of the reversible activation process in MNV. We propose that this reversible activation may have evolved to leverage the extreme conditions in the gut to avoid immune recognition. The gut has a low pH with high concentrations of metal ions and bile salts. Each of these conditions causes the collapse of the P domain onto the shell (red virion) and buries epitopes at the tip while enhancing receptor binding (5, 29, 30). In the epithelium, passive transport of bile salts and metals allows for enhanced infection, while simultaneously bile salts dampen the immune response (43). As the virus drains into the lymphatic system, those metabolites dissipate, and the virus adopts the apo structure (blue virion). The immune system therefore only sees this apo structure and therefore does not recognize the activated form in the gut. In this way, the virus can reinfect the gut in subsequent challenges without requiring escape mutations to avoid the antibody response. However, since the immune system recognizes the apo form, extraintestinal sites are protected in subsequent challenges.

Most viruses exist at a precipice where some environmental trigger and/or receptor starts a cascade of irreversible conformational changes initiating the infection process. Similarly, MNV responds to the low pH and high bile and metal ion concentrations to undergo the transition to the activated state that is optimized for receptor binding. Unlike other viruses, however, this process is wholly reversible. We propose that MNV has developed this as a unique mechanism for immune evasion, and these allosteric escape mutants are mimicking this process. Unlike most viruses, MNV infects the gut tissue where conditions are completely different from those at any other location *in vivo*. Rather than just surviving the trip through the alimentary canal, MNV utilizes these extreme conditions to completely alter its capsid conformation. In this way, without the need for escape mutations, it can evade the antibody response while in the gut. This model is detailed in Fig. 10. Within the gut, MNV is in the activated state (red particles) and enters the epithelium via the M cells. Bile enters the small intestinal tissue via passive transport, while calcium and magnesium are absorbed by the epithelium via paracellular and transcellular transport. Therefore, there are significant concentration gradients of metal ions and bile salts in the tissue directly underneath the intestinal epithelium. This allows for increased infectivity of CD300lf-expressing cells (e.g., macrophages and dendritic cells), promoting replication and shedding. As the virus and infected cells drain from the epithelium, bile and metal ions are reabsorbed, and the virus reverts to the apo state. Therefore, what is presented to the lymphatic system is the apo form of MNV with its open A'B’/E’F’ loop conformation and raised P domain. In this way, the virus can repeatedly infect the gut while being effectively neutralized in the circulation. Importantly, this can explain why infection with high doses of MNV does not protect the gut from subsequent challenge but does protect the spleen where conditions keep MNV in the apo form (15). It seems likely that during the initial response to infection, the high concentrations of antibodies to apo MNV might afford some protection in the gut, but it becomes prone to subsequent infection as the antibody production wanes. Added to this, bile acids are well-known to induce a tolerogenic adaptive T- and B-cell response (41). It is tempting to speculate that bile suppresses the generation of neutralizing antibodies to the activated form of the virus, thus exacerbating the immunological blind spot for activated MNV. Interestingly, a recent study showed that mice with higher levels of bile in the gut have concomitantly higher MNV disease scores (42), making an *in vivo* connection between bile salts and infection. This dynamic and aggressive immune avoidance is only possible because of the extreme environmental differences between the gut and the circulation and the fact that MNV has an extremely flexible capsid where relatively small energy inputs can trigger complete remodeling of the capsid. Since HNoVs similarly have a contracted conformation at low pH (e.g. (1)) and are in the expanded state (apo) in PBS just like MNV (27), it will be interesting to determine how well these results translate to HNoVs. For example, some studies have suggested that adaptive immunity to HNoVs is transient, wherein human volunteers infected with norovirus had symptomatic infections when challenged with the same stock 6 months later.

Perhaps the largest potential impact of these findings is on vaccine development. Because of this rather clever means to avoid immune recognition in the gut, it is necessary to consider these potential conformational changes in the HNoV vaccine design. Theoretically, immunization with a perpetually activated form of the norovirus capsid would be anticipated to result in better protection against infection. Future studies on the MNV animal model will be important to test this concept.

## MATERIALS AND METHODS

### Antibody production and purification

All antibodies were grown in 1 L Wheaton CELLine Flasks. RPMI media without serum was used in the 1 L bulk chamber, and Gibco Cell MAb Medium (animal component free, Cat. 220513) that contained necessary growth factors was used for the 15 mL cell compartment. Approximately twice a week, 15 mL of cells and media were harvested from the cell compartment and replaced with MAb medium. The cells were removed by centrifugation for 10 min at 10,000 *g*. To the supernatant, an equal volume of saturated ammonium sulfate was added, and the mixture was stored at 4°C.

Monoclonal antibody A6.2 is an IgG and therefore purified using a protein G column. Since protein G is relatively insensitive to salt concentrations, the antibody was purified directly from the ammonium sulfate precipitate. The precipitated A6.2 was centrifuged at 10,000 *g* for 10 min, and the pellet was resuspended in ~10 x the volume of the pellet in PBS buffer. The sample was then loaded onto the protein G column, washed extensively with PBS buffer, and eluted with 0.1M glycine buffer, pH 2.7. The low pH of the elution was immediately neutralized using a 1M Tris buffer, pH 8.0. If not used immediately, the antibody was again precipitated with ammonium sulfate.

Monoclonal antibodies 4F9 and 2D3 are IgA antibodies and therefore could not be purified with the protein G column. For these, the precipitated mixtures from the CELLine flasks were centrifuged and resuspended in PBS as above. Approximately 10mL of this resuspension was loaded onto a Cytiva XK-50 column (300 mm x 50 mm i.d.) filled with Sephacryl S-300 media. Fractions containing antibody were pooled and stored in ammonium sulfate. The precipitate was collected by centrifugation (10,000 *g* for 10 min) and dialyzed against 25 mM Tris buffer, pH 7.4. The protein was then purified using a Mono-Q 5/50 Gl anion exchange column on an AKTA system. Buffer A was the same as used for dialysis, while buffer B had an additional 1 M NaCl. Using a gradient of 1% buffer B/mL, the protein eluted at 0.1–0.15 M NaCl. The antibody fractions were pooled and stored as an ammonium sulfate precipitate.

Purity for all antibodies was monitored with SDS-PAGE. Immediately before use, purified antibodies were harvested from the ammonium sulfate precipitate and dialyzed against PBS. Concentrations were determined using an extinction coefficient at 280 nm of 1 mg/mL*OD. For the experiments comparing the relative efficacy, the antibody concentrations were normalized to each other by analyzing the SDS-PAGE using ImageJ (44) and integrating the heavy-chain bands.

### Virus production and purification

The V339I and D348E mutants of MNV-1 were produced and purified as previously described (5). In brief, BV-2 (RRID:CVCL_0182) cells were grown in 4 L spinner flasks in Media B (Gibco S-MEM, 0.1% [wt/vol] Kolliphore P188, 1% vol/vol NEAA, 0.062 g/L penicillin, 0.4 g/L streptomycin sulfate, and 26 mM sodium bicarbonate) until they reached a density of ~0.5–1.0×10^6^ cells/ml. Approximately 6 liters of cells were harvested by centrifuging 4,000 *g* for 10 min. The cells were suspended in 1 L HEPES (AH) media, placed into a 4 L flask, and ~1×10^9^ pfus of MNV was added. Importantly, we found that both mutations reverted to wt after several passages as we scaled up the production. To prevent this, ~10 µg/mL of purified 2D3, 4F9, or A6.2 was added to the infection. The suspension was transferred to a 37°C incubator without CO_2_ and shaken at ~70 rpm in the dark. Mutant replication was apparently slower than that wt, in that the infected cells required 48 hours of incubation for maximal titer compared to 24 hours for wt. The infected cell suspension was centrifuged for 30 min at 5,000 *g,* and the supernatant was collected. To the supernatant, dry NaCl and PEG 8,000 were added to yield 0.3M and 10%, respectively. The solution was then mixed at 4°C overnight. The solution was then centrifuged for 30 min at 5,000 *g,* and the pellet was resuspended in 50–80 mL of PBS. After the suspension was incubated at 4 ° C for several hours, the debris was removed by centrifugation at 10,000 *g* for 30 min. To the supernatant, glycerol was added to a final concentration of 10% (vol/vol) as a cryoprotectant, divided into 1.5 mL aliquots, and stored at −80°C.

Immediately before the cryo-EM experiments, 50–100 mL of this material was thawed and centrifuged at 45,000 rpm for 2 hours in a 50.2 Ti Beckman rotor. The pellets were resuspended in less than a total volume of 3 mL of PBS and allowed to incubate for several hours at 4°C. The debris was removed by centrifugation, and the supernatant was then layered onto Beckman SW41 tubes containing 7.5%–45% linear sucrose gradients using PBS as buffer. After centrifugation for 1.5–2.0 hours at 35,000 rpm at 4°C, MNV forms a band ~two-thirds of the way down the tube. The virus was collected via puncturing the side of the tube with a syringe. The pooled bands were dialyzed overnight against buffer containing 30 mM HEPES, pH 7.4, and 100 mM NaCl. When the titer of the frozen virus was less than 10^7^ pfu/mL, the virus band was often difficult to see. A large section of the gradient near the expected location was collected, the virus pelleted as above, and the sample reseparated on a sucrose gradient. The act of pelleting forces some of the cellular contaminants into a pellet that could not be resuspended. A portion of the virus was used to confirm the presence of the mutation using RT-PCR and Sanger sequencing, as previously described (45). The virus was pelleted using a Beckman 50.2 Ti rotor for 1.5 hours at 45,000 rpm at 4°C and resuspended in the same buffer. The virus was divided into three different portions to which nothing, 1 mM CaCl_2_ (final concentration), or 10 mM GCDCA (final concentration) was added. The samples were immediately frozen for cryo-EM studies.

### Antibody neutralization assays

Virus neutralization plaque assays were performed as previously described (31). BV2 cells from the spinner cultures were added to 6-well tissue culture plates (9.5 cm^2^ surface area) and allowed to attach for ~2 hours. The excess media was removed, and 0.5 mL of virus diluted to ~10^3^–10^8^ pfu/mL with Media B. For neutralization, purified monoclonal A6.2 was added to a final concentration of ~50 µg/mL and incubated with the virus for ~1 hour. To each well, 0.5 mL of diluted virus (+/– antibody) was added, and the virus was allowed to attach for 45 min on a tilting shaker. After incubation, the media was removed, and 2 mL of 50% (vol/vol) low-melting temperature agar/P6 media (31 mM BSA, 79 mM magnesium chloride hexahydrate, Gibco MEM, 2% [vol/vol] NEAA, 0.124 g/L penicillin, 0.8 g/L streptomycin sulfate, and 52 mM sodium bicarbonate) was added to the wells. Once the agar was solidified, 5 mL of Media B was added to each well. The plates were incubated at 37°C and 5% CO2_2_ for 37 hours. After incubation, the media and agar were removed, and a 0.1% crystal violet/20% ethanol mixture was added for plaque visualization. Since the various virus samples had different titers, the neutralization efficacy was calculated by the number of plaques without antibody divided by the plaque count with the antibody added.

### Cryo-electron microscopy (cryo-EM)

MNV samples were at concentrations of ~1 mg/mL. The virus was vitrified as previously described (46) on carbon holey film (R2 × 1 Quantifoil; Micro Tools GmbH, Jena, Germany) grids. Briefly, grids were cleaned in Gatan 950 Solarus plasma cleaner for 40 s in hydrogen-oxygen gas mixture. Four microliters of the V339I or D348E virus solutions was applied to the holey films, blotted with a filter paper, and plunged into liquid ethane. The EM-GP2 (Leica) automated plunger was used for vitrification.

The grids were screened for ice and sample quality and imaged in a Titan Krios G3i (Thermo-Fisher) microscope. The microscope was equipped with a BioQuantum electron energy filter (Ametek, Inc.) and operated at 300 keV. A slit width of 20 eV was used for data collection. Images were acquired with SerialEM (47) using the fast acquisition mode with beam-image shift used for hole centering instead of stage movement. The direct detector camera (K3, Ametek) operated in the counted mode, images were recorded with an overall electron dose of 40 electrons/ Å^2^; the defocus range was −1.5 to −2.5 µm. The pixel size was 1.096Å on the specimen scale.

The data collection statistics are summarized in Table 1.

**TABLE 1:** Data and refinement statistics for the four structures

	V339I Apo	V339I + 2mM Ca^2+^	V339I + 10mM GCDCA	D348E Apo
RCSB	9EAQ	9EAO	9EAP	9EAN
EMD	47840	47838	47839	47837
Instrument used	Titan Krios G3i	Titan Krios G3i	Titan Krios G3i	Titan Krios G3i
No. of images	7,658	7,301	7,892	21,903
No. of particles used	145,934	164,236	209,918	187,851
Software	Cryosparc v4.6	Cryosparc v4.6	Cryosparc v4.6 v4.6	Cryosparc v4.6
Resolution	2.7 Å	2.6 Å	2.7 Å	2.4 Å
No. of protein atoms	11,793	12,069	11,885	11,793
No. of GCDCA atoms	0	0	96	0
No. of calcium atoms	0	3	0	0
CC protein masked	0.86	0.84	0.82	0.81
CC ligand		0.65	0.47	
Ramachandran plot				
Outliers	0.13%	0.2%	0.13%	0.13%
Allowed	4.8%	5.6%	7.9%	5.3%
Favored	95.0%	94.2%	92.0%	94.6%
Geometry deviation				
Bond	0.002Å	0.003Å	0.004Å	0.006Å
Angle	0.57°	0.62°	0.73°	0.83°
B values (min/max/mean)				
Protein	0.0/130/29.7 Å^2^	0.0/66.3/16.3 Å^2^	71.1/277/153 Å^2^	0.0/77.2/24.3
Metal ion		47.2/82.2/60.0 Å^2^		
GCDCA			182/194/227 Å^2^	

### Image processing

For the MNV reconstructions, 7,658, 7,301, 7,892, and 21,903 images were used for the apo V339I, V339I + calcium, V339I + GCDCA, and apo D348E samples that yielded 145,934, 164,236, 209,918, and 187,851 particles, respectively. Our model from our previous image reconstructions (e.g., 30, 31) was used to calculate 25 templates, and particles were picked using the Cryosparc 4.6 (48) template picker. Particles were culled using 2D classification. A low-pass filter was applied to our previous structure, and the non-uniform refinement algorithm yielded a cryo-EM density with Gold Standard Fourier shell resolution of ~2.7Å for all three V339I reconstructions and 2.4Å for the D348E reconstruction.

### Structure refinement

All structure refinement procedures were performed using PHENIX (49). The density of all four reconstructions had the activated conformation where the P domain had contracted onto the shell, as previously observed in the MNV/bile complex (5), and therefore that structure was used as an initial model for building and refinement in PHENIX. To ensure that the model of the icosahedral asymmetric unit did not move into the density of adjacent subunits, the model used for real space refinement included neighboring subunits that formed the A/B and C/C dimers. The model was manually fitted into the EM envelope, and several cycles of real space refinement (rigid body, global minimization, and simulated annealing) in PHENIX (49) and rebuilding in COOT (50) were performed. The final refinement statistics are shown in Table 1.

### Molecular dynamics simulations

The initial structure used for the MD simulation was the x-ray structure of the apo P domain, PDBID: 3LQ6, with a resolution of 2.0Å. This structure has an asymmetric conformation with the A’B’/E’F’ loops of chain A in the “open” conformation and chain B in the “closed” conformation (12). The V339I model was modeled using the Mutate option in VMD (51). Hydrogen atoms were added, using a protonation state of pH 7.0, and each chain was capped with acetylated N-termini and N-methylamidated C-termini using PSFGEN (51). The system was then solvated with TIP3P water with at least 20Å between the protein and the box edge. The system was neutralized, and NaCl ions were added for a salt concentration of 0.11M.

The molecular dynamics simulations were performed with version 2.14 of NAMD (52) with the CHARMM36m force-field parameters (53). Initially, the energy was minimized using the conjugate gradient algorithm for 20K steps and then heated slowly for 25K steps from 0K to 300K with no restraints. Next, the protein atoms were constrained, and the solvent and ions were allowed to equilibrate for 3 ns in the isothermal-isobaric (NPT) ensemble. The constraints on the protein atoms were subsequently removed, and all the atoms were allowed to move for an additional 5 ns of equilibration. A further 900 ns was simulated using the isothermal-isobaric ensemble at a temperature of 300 K and 1 atm. A 2 fs timestep is used to integrate the equations of motion for the 900 ns of production. Particle-mesh Ewald (54) was used to calculate the long-range electrostatic interactions, and van der Waals interactions were truncated at 12Å. All bonds were constrained with the RATTLE algorithm (55). Trajectory frames were saved every 2 ps.

Analyses of the simulation data were performed with CPPTRAJ (56), VMD (51), and PyMol (57) for the residues of the P domain only, i.e., residues 237–522 of each chain. For the root-mean-square deviation (RMSD) calculations, each snapshot was first aligned to the first frame after equilibration, considering only the protein backbone atoms. RMSD values were then calculated for all atoms, except for hydrogens for each chain, A and B, and each domain, P1 (residues 416–522) and P2 (residues 237–415). Mass-weighted radius of gyration (R_g_) was calculated for all non-hydrogen atoms of each domain, P1 and P2.

## Data Availability

The cryo-EM maps for V339I api, V339I + 2mM Ca^2+^, V339I + 10mM GCDCA, and D348E apo are available in the EM database as 47840, 47838, 47839, and 47837, respectively, and the corresponding atomic models are in the PDB database as 9EAQ, 9EAO, 9EAP, and 9EAN (https://www.rcsb.org).

## SUPPLEMENTAL MATERIAL

Figure S1 jvi.00219-25-s0001.docx Example of loop density.

Figure S2 jvi.00219-25-s0002.docx Radius of gyration during dynamics simulations.

Figure S3 jvi.00219-25-s0003.docx Root mean square deviations during simulations.

Figure S4 jvi.00219-25-s0004.docx Dimer rotation during simulations.

Video S5 jvi.00219-25-s0005.mov AB EF loop dynamics of wt P domain during simulations.

Video S6 jvi.00219-25-s0006.mov Motion of the outer loops of the V339I mutant MNV P domain during dynamics simulations.

Video S7 jvi.00219-25-s0007.mov Animation showing the overall location of V339I corresponding to Fig. 7A.

Video S8 jvi.00219-25-s0008.mov Closeup of V339I corresponding to Fig. 7B.

Video S9 jvi.00219-25-s0009.mov C'D' loop of D348E in hypothetical apo conformation corresponding to Fig. 8E.

Video S10 jvi.00219-25-s0010.mov C'D' loop of D348E mutant in activated state corresponding to Fig. 8F.

ASM does not own the copyrights to Supplemental Material that may be linked to, or accessed through, an article. The authors have granted ASM a non-exclusive, world-wide license to publish the Supplemental Material files. Please contact the corresponding author directly for reuse.

## References

[1] Prasad BVV, Hardy ME, Dokland T, Bella J, Rossmann MG, Estes MK. 1999. X-ray crystallographic structure of the Norwalk virus capsid. Science 286:287–290. doi:10.1126/science.286.5438.287 10514371

[2] Prasad BV, Hardy ME, Jiang X, Estes MK. 1996. Structure of Norwalk virus. Arch Virol Suppl 12:237–242. doi:10.1007/978-3-7091-6553-9_25 9015120

[3] Prasad BV, Matson DO, Smith AW. 1994. Three-dimensional structure of calicivirus. J Mol Biol 240:256–264. doi:10.1006/jmbi.1994.1439 8028008

[4] Katpally U, Wobus CE, Dryden K, Virgin HWI, Smith TJ. 2008. Structure of antibody-neutralized murine norovirus and unexpected differences from viruslike particles. J Virol 82:2079–2088. doi:10.1128/JVI.02200-07 18094184 PMC2258928

[5] Sherman MB, Williams AN, Smith HQ, Nelson C, Wilen CB, Fremont DH, Virgin HW, Smith TJ. 2019. Bile salts alter the mouse norovirus capsid conformation: possible implications for cell attachment and immune evasion. J Virol 93:e00970-19. doi:10.1128/JVI.00970-19 31341042 PMC6744230

[6] Choi J-M, Hutson AM, Estes MK, Prasad BVV. 2008. Atomic resolution structural characterization of recognition of histo-blood group antigens by Norwalk virus. Proc Natl Acad Sci USA 105:9175–9180. doi:10.1073/pnas.0803275105 18599458 PMC2453692

[7] Tan M, Hegde RS, Jiang X. 2004. The P domain of norovirus capsid protein forms dimer and binds to histo-blood group antigen receptors. J Virol 78:6233–6242. doi:10.1128/JVI.78.12.6233-6242.2004 15163716 PMC416535

[8] Nelson CA, Wilen CB, Dai Y-N, Orchard RC, Kim AS, Stegeman RA, Hsieh LL, Smith TJ, Virgin HW, Fremont DH. 2018. Structural basis for murine norovirus engagement of bile acids and the CD300lf receptor. Proc Natl Acad Sci U S A 115:E9201–E9210. doi:10.1073/pnas.1805797115 30194229 PMC6166816

[9] Kilic T, Koromyslova A, Malak V, Hansman GS. 2018. Atomic structure of the murine norovirus protruding domain and soluble CD300lf receptor complex. J Virol 92:e00413-18. doi:10.1128/JVI.00413-18 29563286 PMC5952153

[10] Donaldson EF, Lindesmith LC, Lobue AD, Baric RS. 2010. Viral shape-shifting: norovirus evasion of the human immune system. Nat Rev Microbiol 8:231–241. doi:10.1038/nrmicro2296 20125087 PMC7097584

[11] Nilsson M, Hedlund KO, Thorhagen M, Larson G, Johansen K, Ekspong A, Svensson L. 2003. Evolution of human calicivirus RNA in vivo: accumulation of mutations in the protruding P2 domain of the capsid leads to structural changes and possibly a new phenotype. J Virol 77:13117–13124. doi:10.1128/jvi.77.24.13117-13124.2003 14645568 PMC296070

[12] Taube S, Rubin JR, Katpally U, Smith TJ, Kendall A, Stuckey JA, Wobus CE. 2010. High-resolution X-ray structure and functional analysis of the murine norovirus 1 capsid protein protruding domain. J Virol 84:5695–5705. doi:10.1128/JVI.00316-10 20335262 PMC2876589

[13] Kolawole AO, Smith HQ, Svoboda SA, Lewis MS, Sherman MB, Lynch GC, Pettitt BM, Smith TJ, Wobus CE. 2017. Norovirus escape from broadly neutralizing antibodies is limited to allostery-like mechanisms. mSphere 2:e00334-17. doi:10.1128/mSphere.00334-17 29062895 PMC5646240

[14] Johnson PC, Mathewson JJ, DuPont HL, Greenberg HB. 1990. Multiple-challenge study of host susceptibility to Norwalk gastroenteritis in US adults. J Infect Dis 161:18–21. doi:10.1093/infdis/161.1.18 2153184

[15] Liu G, Kahan SM, Jia Y, Karst SM. 2009. Primary high-dose murine norovirus 1 infection fails to protect from secondary challenge with homologous virus. J Virol 83:6963–6968. doi:10.1128/JVI.00284-09 19403675 PMC2698529

[16] Karst SM. 2010. Pathogenesis of noroviruses, emerging RNA viruses. Viruses 2:748–781. doi:10.3390/v2030748 21994656 PMC3185648

[17] Hu L, Salmen W, Chen R, Zhou Y, Neill F, Crowe JE, Atmar RL, Estes MK, Prasad BVV. 2022. Atomic structure of the predominant GII.4 human norovirus capsid reveals novel stability and plasticity. Nat Commun 13:1241. doi:10.1038/s41467-022-28757-z 35273142 PMC8913647

[18] Prasad BVV, Atmar RL, Ramani S, Palzkill T, Song Y, Crawford SE, Estes MK. 2025. Norovirus replication, host interactions and vaccine advances. Nat Rev Microbiol. doi:10.1038/s41579-024-01144-9 PMC12088900 39824927

[19] Helm EW, Peiper AM, Phillips M, Williams CG, Sherman MB, Kelley T, Smith HQ, Jacobs SO, Shah D, Tatum SM, Iyer N, Grodzki M, Morales Aparicio JC, Kennedy EA, Manzi MS, Baldridge MT, Smith TJ, Karst SM. 2022. Environmentally-triggered contraction of the norovirus virion determines diarrheagenic potential. Front Immunol 13:1043746. doi:10.3389/fimmu.2022.1043746 36389732 PMC9664153

[20] Agus SG, Dolin R, Wyatt RG, Tousimis AJ, Northrup RS. 1973. Acute infectious nonbacterial gastroenteritis: intestinal histopathology. Histologic and enzymatic alterations during illness produced by the Norwalk agent in man. Ann Intern Med 79:18–25. doi:10.7326/0003-4819-79-1-18 4721173

[21] Dolin R, Levy AG, Wyatt RG, Thornhill TS, Gardner JD. 1975. Viral gastroenteritis induced by the Hawaii agent. Am J Med 59:761–768. doi:10.1016/0002-9343(75)90461-1 811119

[22] Karandikar UC, Crawford SE, Ajami NJ, Murakami K, Kou B, Ettayebi K, Papanicolaou GA, Jongwutiwes U, Perales MA, Shia J, Mercer D, Finegold MJ, Vinjé J, Atmar RL, Estes MK. 2016. Detection of human norovirus in intestinal biopsies from immunocompromised transplant patients. J Gen Virol 97:2291–2300. doi:10.1099/jgv.0.000545 27412790 PMC5756488

[23] Schreiber DS, Blacklow NR, Trier JS. 1973. The mucosal lesion of the proximal small intestine in acute infectious nonbacterial gastroenteritis. N Engl J Med 288:1318–1323. doi:10.1056/NEJM197306212882503 4706274

[24] Katpally U, Voss NR, Cavazza T, Taube S, Rubin JR, Young VL, Stuckey J, Ward VK, Virgin HW IV, Wobus CE, Smith TJ. 2010. High-resolution cryo-electron microscopy structures of murine norovirus 1 and rabbit hemorrhagic disease virus reveal marked flexibility in the receptor binding domains. J Virol 84:5836–5841. doi:10.1128/JVI.00314-10 20335264 PMC2876586

[25] Kolawole AO, Li M, Xia C, Fischer AE, Giacobbi NS, Rippinger CM, Proescher JBG, Wu SK, Bessling SL, Gamez M, Yu C, Zhang R, Mehoke TS, Pipas JM, Wolfe JT, Lin JS, Feldman AB, Smith TJ, Wobus CE. 2014. Flexibility in surface-exposed loops in a virus capsid mediates escape from antibody neutralization. J Virol 88:4543–4557. doi:10.1128/JVI.03685-13 24501415 PMC3993751

[26] Kolawole AO, Xia C, Li M, Gamez M, Yu C, Rippinger CM, Yucha RE, Smith TJ, Wobus CE. 2014. Newly isolated mAbs broaden the neutralizing epitope in murine norovirus. J Gen Virol 95:1958–1968. doi:10.1099/vir.0.066753-0 24899153 PMC4135088

[27] Hansman GS, Taylor DW, McLellan JS, Smith TJ, Georgiev I, Tame JRH, Park S-Y, Yamazaki M, Gondaira F, Miki M, Katayama K, Murata K, Kwong PD. 2012. Structural basis for broad detection of genogroup II noroviruses by a monoclonal antibody that binds to a site occluded in the viral particle. J Virol 86:3635–3646. doi:10.1128/JVI.06868-11 22278249 PMC3302548

[28] Smith HQ, Smith TJ. 2019. The dynamic capsid structures of the noroviruses. Viruses 11:235. doi:10.3390/v11030235 30857192 PMC6466125

[29] Sherman M, Cox F, Smith H, Habib MH, Karst S, Wobus CE, Smith TJ. 2024. The reversible activation of norovirus by metal ions. J Virol 98:e0173523. doi:10.1128/jvi.01735-23 38236007 PMC10878237

[30] Williams AN, Sherman MB, Smith HQ, Taube S, Pettitt BM, Wobus CE, Smith TJ. 2021. Multiple signals in the gut contract the mouse norovirus capsid to block antibody binding while enhancing receptor affinity. J Virol 95:e0147121. doi:10.1128/JVI.01471-21 34468172 PMC8549501

[31] Williams AN, Sherman MB, Smith HQ, Taube S, Pettitt BM, Wobus CE, Smith TJ. 2021. A norovirus uses bile salts to escape antibody recognition while enhancing receptor binding. J Virol 95:e0017621. doi:10.1128/JVI.00176-21 33827952 PMC8315966

[32] Sherman MB, Williams AN, Smith HQ, Pettitt BM, Wobus CE, Smith TJ. 2021. Structural studies on the shapeshifting murine norovirus. Viruses 13:2162. doi:10.3390/v13112162 34834968 PMC8621758

[33] Chen R, Neill JD, Noel JS, Hutson AM, Glass RI, Estes MK, Prasad BVV. 2004. Inter- and intragenus structural variations in caliciviruses and their functional implications. J Virol 78:6469–6479. doi:10.1128/JVI.78.12.6469-6479.2004 15163740 PMC416503

[34] Ossiboff RJ, Zhou Y, Lightfoot PJ, Prasad BVV, Parker JSL. 2010. Conformational changes in the capsid of a Calicivirus upon interaction with its functional receptor. J Virol 84:5550–5564. doi:10.1128/JVI.02371-09 20357100 PMC2876613

[35] Sherman MB, Williams AN, Smith HQ, Wobus CE, Smith TJ. 2022. Mouse norovirus uses host metabolites to enhance receptor binding and evade immune recognition. MRAJ 10. doi:10.18103/mra.v10i11.3270 PMC13464545 42592225

[36] Wobus CE, Karst SM, Thackray LB, Chang K-O, Sosnovtsev SV, Belliot G, Krug A, Mackenzie JM, Green KY, Virgin HW. 2004. Replication of norovirus in cell culture reveals a tropism for dendritic cells and macrophages. PLoS Biol 2:e432. doi:10.1371/journal.pbio.0020432 15562321 PMC532393

[37] Lochridge VP, Hardy ME. 2007. A single-amino-acid substitution in the P2 domain of VP1 of murine norovirus is sufficient for escape from antibody neutralization. J Virol 81:12316–12322. doi:10.1128/JVI.01254-07 17804495 PMC2168968

[38] Smith TJ, Chase ES, Schmidt TJ, Olson NH, Baker TS. 1996. Neutralizing antibody to human rhinovirus 14 penetrates the receptor-binding canyon. Nature New Biol 383:350–354. doi:10.1038/383350a0 PMC4167671 8848050

[39] Che Z, Olson NH, Leippe D, Lee WM, Mosser AG, Rueckert RR, Baker TS, Smith TJ. 1998. Antibody-mediated neutralization of human rhinovirus 14 explored by means of cryoelectron microscopy and X-ray crystallography of virus-Fab complexes. J Virol 72:4610–4622. doi:10.1128/JVI.72.6.4610-4622.1998 9573224 PMC109976

[40] Smith TJ. 2011. Structural studies on antibody recognition and neutralization of viruses. Curr Opin Virol 1:150–156. doi:10.1016/j.coviro.2011.05.020 21887208 PMC3163491

[41] Su X, Gao Y, Yang R. 2023. Gut microbiota derived bile acid metabolites maintain the homeostasis of gut and systemic immunity. Front Immunol 14:1127743. doi:10.3389/fimmu.2023.1127743 37256134 PMC10225537

[42] Peiper AM, Aparicio JM, Phophi L, Hu Z, Helm EW, Phillips M, Williams CG, Subramanian S, Cross M, Iyer N, Nguyen Q, Newsome R, Jobin C, Langel SN, Bucardo F, Becker-Dreps S, Tan X-D, Dawson PA, Karst SM. 2024. Metabolic immaturity of newborns and breast milk bile acid metabolites are the central determinants of heightened neonatal vulnerability to norovirus diarrhea. bioRxiv. doi:10.1101/2024.05.01.592031 PMC11392616 39214086

[43] Sun R, Xu C, Feng B, Gao X, Liu Z. 2021. Critical roles of bile acids in regulating intestinal mucosal immune responses. Therap Adv Gastroenterol 14:17562848211018098. doi:10.1177/17562848211018098 34104213 PMC8165529

[44] Schneider CA, Rasband WS, Eliceiri KW. 2012. NIH Image to ImageJ: 25 years of image analysis. Nat Methods 9:671–675. doi:10.1038/nmeth.2089 22930834 PMC5554542

[45] Hsu CC, Wobus CE, Steffen EK, Riley LK, Livingston RS. 2005. Development of a microsphere-based serologic multiplexed fluorescent immunoassay and a reverse transcriptase PCR assay to detect murine norovirus 1 infection in mice. Clin Diagn Lab Immunol 12:1145–1151. doi:10.1128/CDLI.12.10.1145-1151.2005 16210475 PMC1247840

[46] Sherman MB, Guenther RH, Tama F, Sit TL, Brooks CL, Mikhailov AM, Orlova EV, Baker TS, Lommel SA. 2006. Removal of divalent cations induces structural transitions in red clover necrotic mosaic virus, revealing a potential mechanism for RNA release. J Virol 80:10395–10406. doi:10.1128/JVI.01137-06 16920821 PMC1641784

[47] Mastronarde DN. 2005. Automated electron microscope tomography using robust prediction of specimen movements. J Struct Biol 152:36–51. doi:10.1016/j.jsb.2005.07.007 16182563

[48] Punjani A, Rubinstein JL, Fleet DJ, Brubaker MA. 2017. cryoSPARC: algorithms for rapid unsupervised cryo-EM structure determination. Nat Methods 14:290–296. doi:10.1038/nmeth.4169 28165473

[49] Afonine PV, Grosse-Kunstleve RW, Adams PD. 2005. The Phenix refinement framework. CCP4 Newsletter 42:Contribution 8.

[50] Emsley P, Cowtan K. 2004. Coot: model-building tools for molecular graphics. Acta Cryst D 60:2126–2132. doi:10.1107/S0907444904019158 15572765

[51] Humphrey W, Dalke A, Schulten K. 1996. VMD: visual molecular dynamics. J Mol Graph 14:33–38, doi:10.1016/0263-7855(96)00018-5 8744570

[52] Phillips JC, Braun R, Wang W, Gumbart J, Tajkhorshid E, Villa E, Chipot C, Skeel RD, Kalé L, Schulten K. 2005. Scalable molecular dynamics with NAMD. J Comput Chem 26:1781–1802. doi:10.1002/jcc.20289 16222654 PMC2486339

[53] Huang J, Rauscher S, Nawrocki G, Ran T, Feig M, de Groot BL, Grubmüller H, MacKerell AD Jr. 2017. CHARMM36m: an improved force field for folded and intrinsically disordered proteins. Nat Methods 14:71–73. doi:10.1038/nmeth.4067 27819658 PMC5199616

[54] Essmann U, Perera L, Berkowitz ML, Darden T, Lee H, Pedersen LG. 1995. A smooth particle mesh Ewald method. J Chem Phys 103:8577–8593. doi:10.1063/1.470117

[55] Andersen HC. 1983. Rattle: a “velocity” version of the shake algorithm for molecular dynamics calculations. J Comput Phys 52:24–34. doi:10.1016/0021-9991(83)90014-1

[56] Roe DR, Cheatham TE III. 2018. Parallelization of CPPTRAJ enables large scale analysis of molecular dynamics trajectory data. J Comput Chem 39:2110–2117. doi:10.1002/jcc.25382 30368859 PMC7313716

[57] The PyMOL molecular graphics system Vrp. Schrödinger.

