# Supplementary material for: Murine norovirus allosteric escape mutants mimic gut activation
Source: J Virol. 2025 May 12;99(6):e00219-25. doi: 10.1128/jvi.00219-25 (PMC12172446; doi:10.1128/jvi.00219-25)
Supplement: Figure S1 — Example of loop density. [file jvi.00219-25-s0001.docx]

Figure S1

Figure S1: Density at the tips of the P domains. While Apo V339I is in the activated conformation, the density of the loops is markedly disordered (top). By adding GCDCA, the activation process is complete, and the loops become far more ordered (Bottom).
