# Supplementary material for: Murine norovirus allosteric escape mutants mimic gut activation
Source: J Virol. 2025 May 12;99(6):e00219-25. doi: 10.1128/jvi.00219-25 (PMC12172446; doi:10.1128/jvi.00219-25)
Supplement: Figure S2 — Radius of gyration during dynamics simulations. [file jvi.00219-25-s0002.docx]

Figure S2


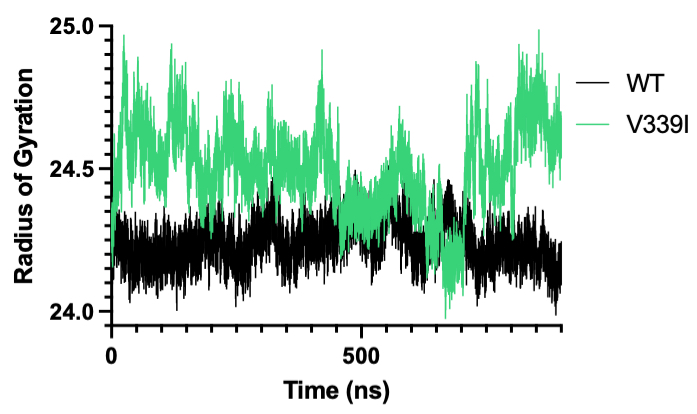


Figure S2: The radius of gyration of wt (black) and V339I (green) during the 900ns simulation. While both structures were stable during the simulation, V339I appeared to be more mobile.
