# Supplementary material for: Murine norovirus allosteric escape mutants mimic gut activation
Source: J Virol. 2025 May 12;99(6):e00219-25. doi: 10.1128/jvi.00219-25 (PMC12172446; doi:10.1128/jvi.00219-25)
Supplement: Figure S3 — Root mean square deviations during simulations. [file jvi.00219-25-s0003.docx]

Figure S3


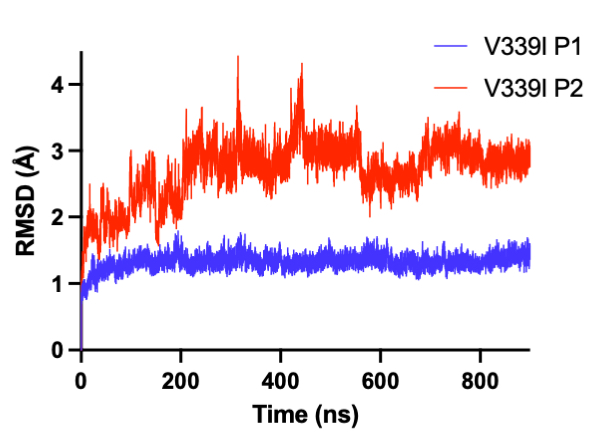


Figure S3: Root-mean-square deviations of apo V339I separated according P1 (blue) and P2 (red) domains. Note that the P2 domain is far more mobile than the P1 domain.
